# Plant Actin Cytoskeleton Dynamics in Salt Stress Tolerance: From Structural Remodeling to Regulatory Networks

**DOI:** 10.3390/plants15213316

**Published:** 2026-10-26

**Authors:** Shuyi Chen, Fansen Zeng, Xinwen Liang, Yutong Qian, Yufeng Wang, Thomas W. Okita, Li Tian, Laining Zhang

**Affiliations:** 1 Collaborative Innovation Center for Efficient and Green Production of Agriculture in Mountainous Areas of Zhejiang Province, College of Horticulture, Zhejiang A&F University, Hangzhou 311300, China; 2 Key Laboratory of Quality and Safety Control for Subtropical Fruit and Vegetable, Ministry of Agriculture and Rural Affairs, College of Horticulture Science, Zhejiang A&F University, Hangzhou 311300, China; 3 Jixian Honors College, Zhejiang A&F University, Hangzhou 311300, China; 4 Institute of Biological Chemistry, Washington State University, Pullman, WA 99164, USA; okita@wsu.edu

**Keywords:** cytoskeleton, actin, actin-binding proteins, salt stress, root growth

## Abstract

Soil salinization causes osmotic stress and ionic toxicity in plant cells and severely restricts crop growth and global agricultural productivity. The actin cytoskeleton functions as a rapidly responsive structural module and core signal integrator, with fast structural remodeling triggered upon salt exposure. This review systematically characterizes salt-induced actin rearrangement profiles and dissects the regulatory functions of key actin-binding proteins (ABPs) in governing actin nucleation, severing, bundling and steady-state turnover under saline conditions. Cross-talk among interconnected signaling cascades, including Ca^2+^ signals, the salt overly sensitive (SOS) pathway, reactive oxygen species (ROS) and abscisic acid (ABA) signaling, that orchestrate actin dynamics is synthesized. Key findings highlight that actin-binding proteins act as central molecular hubs linking upstream stress signaling to downstream adaptive outputs, and that actin remodeling underpins multiple salt-tolerance-related physiological events, such as cellular ion homeostasis, plastic root growth, vesicle trafficking, and the modulation of salt-triggered premature programmed cell death. Beyond actin-specific pathways, reciprocal functional interplays between actin filaments and microtubules jointly fine-tune plant salt acclimation. Unresolved questions are discussed and future research directions are proposed to bridge the gap between cytoskeletal dynamics and practical strategies for developing salt-tolerant crops. Overall, the novelty of this review lies in its salt stress-centered, multi-faceted integration of actin dynamics, from actin itself and its remodeling regulators to signaling crosstalk, physiological outputs, and actin–microtubule interplay. This review constructs a comprehensive theoretical framework linking cytoskeletal dynamics to plant salt adaptation and outlines research directions that may inform future strategies for developing salt-tolerant crops.

## 1. Introduction

Salinity affects over one-third of global irrigated farmland [1], threatening food security by inhibiting seed germination, retarding vegetative growth, impairing reproductive development and reducing final yield [2]. The toxic impacts of salinity span the entire plant life cycle. At germination, salt stress inhibits water uptake by seeds and exerts direct ion toxicity on embryos, delaying and reducing germination [3]. During vegetative growth, the osmotic component disrupts cellular turgor and reduces water absorption, while the ionic component, driven by progressive Na^+^ accumulation, triggers ionic imbalance, oxidative bursts, and severe organelle damage, together impairing photosynthesis and biomass accumulation [4]. The reproductive stage is particularly sensitive, as salinity reduces pollen viability, limits stamen elongation, and causes male sterility, ultimately decreasing seed set and yield [5]. In severely affected regions, salinity stress can reduce crop yields by up to 70% [1].

To counteract saline environments, plants activate multilayered adaptive programs. These include osmotic adjustment via the accumulation of compatible solutes such as proline, glycine betaine, and soluble sugars to maintain cell water potential and turgor pressure [6]; selective ion uptake, compartmentalization, and efflux to restrict excessive Na^+^ accumulation in the cytoplasm and retain essential K^+^ for ion homeostasis [7,8]; enhanced antioxidant defense systems comprising enzymatic and non-enzymatic antioxidants to scavenge excess reactive oxygen species (ROS) and alleviate oxidative damage to membranes, proteins and nucleic acids [9]; and the modulation of stress-related hormone signaling and the reprogramming of stress-responsive gene expression to regulate growth adaptation and stress tolerance [10]. These synergistic physiological and molecular responses enable plants to mitigate salt-induced damage, sustain basic metabolic activities, and maintain growth and productivity under saline conditions.

For decades, research on plant salt tolerance has focused primarily on ion transporters (such as the SOS pathway), osmotic adjustment via compatible solutes, antioxidant defense systems and the underlying transcriptional reprogramming. However, accumulating evidence indicates that the plant cytoskeleton plays an equally crucial but often overlooked role in mediating cellular adaptation to salt stress. Far from being a passive structural scaffold, the cytoskeleton functions as a dynamic sensory hub that perceives and transduces salt-induced signals, orchestrating a suite of adaptive responses ranging from adjusted cell wall architecture and vesicle trafficking to the re-establishment of polarized growth [11]. Salt stress rapidly triggers cytoskeletal reorganization, including depolymerization, severing, and reorientation of filamentous networks, which in turn modulates the activity and subcellular localization of key signaling proteins and ion transporters. This dynamic remodeling is not merely a symptom of stress-induced damage, but rather an active regulatory mechanism that fine-tunes cellular physiology to mitigate ionic and osmotic imbalances.

Among the two major components of the plant cytoskeleton, microtubules (MTs) and actin filaments (AF), the latter has emerged as a particularly prominent player in salt stress responses due to its intimate connection with membrane dynamics, organelle movement, and ROS signaling. A growing body of evidence has established that actin filaments undergo rapid and precise spatiotemporal remodeling upon salt exposure, a process governed by a suite of actin-binding proteins (ABPs) including profilin, villin, actin-depolymerizing factors (ADFs), and formins [12,13]. This dynamic reorganization, which can manifest as fragmentation, bundling, or cortical fine-tuning depending on stress intensity and duration, directly influences multiple facets of salt adaptation. At the cellular level, actin dynamics regulate the activity and plasma membrane retention of key ion transporters such as SOS1 and H^+^-ATPases, thereby modulating Na^+^ extrusion and K^+^/Na^+^ homeostasis [14]. Concurrently, the actin cytoskeleton coordinates vesicle trafficking pathways that facilitate vacuolar compartmentalization of excess Na^+^ and the recycling of stress-resilient membrane proteins [15,16]. Beyond these trafficking functions, actin also serves as a critical node in ROS signaling. Salt-induced ROS bursts both depend on and feed back into actin organization through redox-sensitive ABPs, forming a regulatory loop that coordinates cytoskeletal status with oxidative stress output [9]. Moreover, emerging studies have implicated nuclear actin in stress-responsive gene expression, hinting at a previously unappreciated transcriptional dimension of actin function [17]. Despite these advances, the precise molecular mechanisms by which actin responds to salt-related signals and translates them into adaptive outputs remain incompletely understood, and the interplay between actin and microtubules under saline conditions warrants further investigation.

While earlier reviews have established the fundamental architecture of the actin cytoskeleton and its associated proteins [18], as well as the broad involvement of the plant cytoskeleton in abiotic stress signaling including SOS-Ca^2+^ crosstalk, actin-related protein 2/3 (ARP2/3)-mediated mitochondrial regulation, and RSA3-ROS interactions [13], the specific contribution of actin dynamics to salt acclimation has remained embedded within these broader frameworks. The present review advances beyond these foundations in three key respects. First, we extend the signaling framework by integrating recent discoveries that directly link salt perception to actin remodeling, including the SOS2-RhoGDI1 (Rho GDP Dissociation Inhibitor 1)-ROP2 (Rho-related GTPase from Plants 2) module that couples the canonical SOS pathway to actin-dependent root hair development [19], and the SPIRRIG (SPI) mediated stabilization of BRICK1 (BRK1) that connects membrane trafficking to salt-induced root hair plasticity [20]. Second, we refine earlier views by incorporating studies that have emerged recently, including the identification of Myosin XI-1 as a mediator of salt tolerance through a Na^+^ transport pathway [21], the demonstration that AtFH5, a Class I formin, contributes to seedling salt resistance [22], and the emerging recognition that nuclear actin functions extend beyond cytoskeletal regulation to include transcriptional control under abiotic stress [11]. Third, we identify critical uncertainties that previous reviews did not resolve, notably how actin filaments and microtubules coordinate their responses under salt stress, whether through direct physical coupling or through shared signaling intermediates, and whether actin remodeling is a passive downstream consequence of salt signaling or an active determinant of salt tolerance. By addressing these questions, this review generates a new conceptual framework that actin is not merely one of several cytoskeletal components affected by salinity, but rather a central integration node where ion transport, vesicle trafficking, ROS signaling, and transcriptional regulation converge to shape salt acclimation outcomes.

## 2. *ACTIN* Gene Family in Plants: An Overview

### 2.1. ACTIN Gene Family: Functional Diversification and Genomic Distribution

Actins in plants are encoded by a multigene family, the size of which varies considerably across different species [23]. In the model plant *Arabidopsis thaliana*, the *ACTIN* gene family comprises eight functional members, widely dispersed on four of the five chromosomes, accompanied by two probable pseudogenes [23]. These eight isoforms are phylogenetically divided into two major classes. *ACT2*, *ACT7*, and *ACT8* are predominantly expressed in vegetative tissue, such as roots, stems, and leaves, and thus called vegetative *ACTINs* [24,25], while *ACT1*, *ACT3*, *ACT4*, *ACT11*, and *ACT12* are highly expressed in reproductive tissues and thus called the reproductive *ACTINs* [25,26,27,28]. It was suggested that all expressed *Arabidopsis ACTIN* genes are unlinked [29] and the vegetative and reproductive classes of *ACTIN* have evolved separately [25]. The vegetative class is considered more primitive, whereas the reproductive actins represent a more diverged group, and this diversification is thought to have originated early in the evolution of vascular plants [30]. In *Arabidopsis*, the eight functional isotypes differ at the amino acid level by 0.3–8.8%, with the most distant isotypes ACT2 and ACT4 differing by 33 amino acids. At the nucleotide level, even closely related paralogs differ by 9–12%, and overall sequence divergence reaches up to 22% [23]. Despite this nucleotide variability, the high amino acid sequence conservation suggests extraordinarily strong selective pressure on protein structure.

Beyond *Arabidopsis*, genome-wide surveys have revealed the *ACTIN* gene numbers in a diverse array of plant species. Among monocots, rice (*Oryza sativa*) contains at least eight to ten functional *ACTIN* genes, with a total of 17 actin domain–containing genes identified in the genome [31]. Maize (*Zea mays*) has been reported to contain as many as 21 *ACTIN* family members, though some likely represent pseudogenes [23], while *Sorghum bicolor* contains approximately 10 *ACTIN* genes [23]. In eudicots, the family size is similarly variable. Soybean (*Glycine max*) possesses 18 *ACTIN* family members (*GmACT1*-*GmACT18*) identified by genome-wide analysis [32], poplar (*Populus trichocarpa*) contains 8-9 *ACTIN* genes [23], and grapevine (*Vitis vinifera*) has at least six *ACTIN* genes [23]. In *Brassica napus*, 69 genes encoding actin domain-containing proteins were identified, although only 25 are the conventional *ACTIN* genes encoding 378-amino acid proteins [33]. This family size variability is not unique to angiosperms. Mosses (*Physcomitrium patens*) contain approximately 10 *ACTIN* genes, gymnosperms (*Pinus taeda*) approximately 10, while the green algae *Chlamydomonas reinhardtii* have only 2, suggesting a pattern of gene expansion during land plant evolution [23].

Despite high amino acid sequence conservation, actin isoforms are functionally non-equivalent. Mutant and complementation studies in *Arabidopsis* have demonstrated that vegetative actins cannot fully substitute for reproductive actins, and vice versa, indicating genuine functional specialization [34]. This functional and regulatory diversification underscores the importance of considering individual actin isoforms when investigating their roles in specific stress responses, including salt tolerance.

### 2.2. Stress-Responsive Expression of ACTIN Genes and Implications for Reference Gene Usage

Beyond their functional specialization, many members of the *ACTIN* gene family display stress-responsive expression profiles, implying their potential involvement in environmental adaptation. For instance, the expression of *Arabidopsis ACT7* is induced by drought and osmotic stress [17]. In rice, ten *OsACT* genes that share high sequence similarity with *Arabidopsis ACTIN* genes were studied for their transcriptional responses to cold, salt and drought stresses [31]. Notably, all ten *OsACT* members were markedly upregulated in both leaves and roots upon cold exposure. By contrast, their expression profiles were relatively mild yet statistically significant under salt and drought treatments. Specifically, *OsACT5* and *OsACT6* were persistently upregulated in both tissues under drought stress, while *OsACT9* and *OsACT10* displayed prominent downregulation. In sweet potato, all thirty candidate *ACTIN* genes showed dynamic transcriptional alterations following treatments with 200 mM NaCl, 20% PEG, or 50 μM ABA [35]. Changes in expression of *ACTIN* genes under various environmental stresses were also observed in cotton [36] and *Brassica napus* [33]. These findings demonstrate that *ACTIN* genes are not constitutively expressed, but instead, their transcription is highly sensitive to external environmental cues and internal stimuli. These observations also uncover functional divergence among actin isoforms during abiotic stress responses and establish a functional connection between actin dynamics and stress signaling cascades.

The stress-responsiveness of *ACTIN* genes carries an important methodological implication. The marked induction or repression of *ACTIN* transcripts under various abiotic stresses indicates that they are unsuitable as reference genes for stress-related expression analyses. This cautionary note is further supported by studies in other plant species. While eggplant *ACTIN* was identified as the most stable gene under salt stress among eight candidate reference genes [37], this was not the case in other plant species. In *Lycium barbarum*, *LbACT7* exhibited only moderate expression stability and introduced significant errors under certain specific treatments, such as across different organs and developmental stages [38]. In *Miscanthus sinensis*, *ACTIN* was among the least stable reference genes among the tested 12 candidate reference genes in root tissues under various treatments [39]. These observations collectively indicate that the expression stability of actin genes is highly dependent on species, tissue type, and stress condition, and therefore the indiscriminate use of *ACTIN* as a reference gene in RT-qPCR studies, particularly under salt or other abiotic stresses, should not be assumed but carefully validated.

In summary, plant *ACTIN* genes form a diversified multigene family whose copy number expanded during land-plant evolution. Although actin proteins retain high sequence conservation under strong purifying selection, vegetative- and reproductive-preferential isoforms are functionally non-equivalent, and numerous paralogs display isoform-specific transcriptional changes under abiotic stresses such as salt, cold and drought. Importantly, this stress-responsive transcription means that actin genes cannot be presumed to be stable reference genes for RT-qPCR under stress treatments, and stability validation is required for each experimental system. Collectively, the genomic divergence and stress-sensitive transcription of *ACTIN* genes provide the molecular foundation for cytoskeleton-dependent stress adaptation. Having outlined these genetic features, we next turn to the structural and dynamic properties of actin filaments, the functional executors of actin-dependent cellular events under salt stress.

## 3. The Plant Actin Cytoskeleton: Components and Regulatory Machinery

### 3.1. Overview of Structure and Dynamics of Actin Filaments

Actin is a highly conserved globular monomeric protein (G-actin) of approximately 42 kDa that reversibly polymerizes into helical filamentous structures (AFs, also called F-actin) [40]. In plant cells, AFs form elaborate and highly dynamic arrays that undergo continuous assembly and disassembly, a process essential for their diverse cellular functions [18]. Under optimal growth conditions, AFs are organized into distinct higher-order structures depending on cell type and developmental stage, including cortical actin arrays beneath the plasma membrane, cytoplasmic cables running longitudinally, and specialized arrays in tip-growing cells such as root hairs and pollen tubes [41].

**Figure 1:**
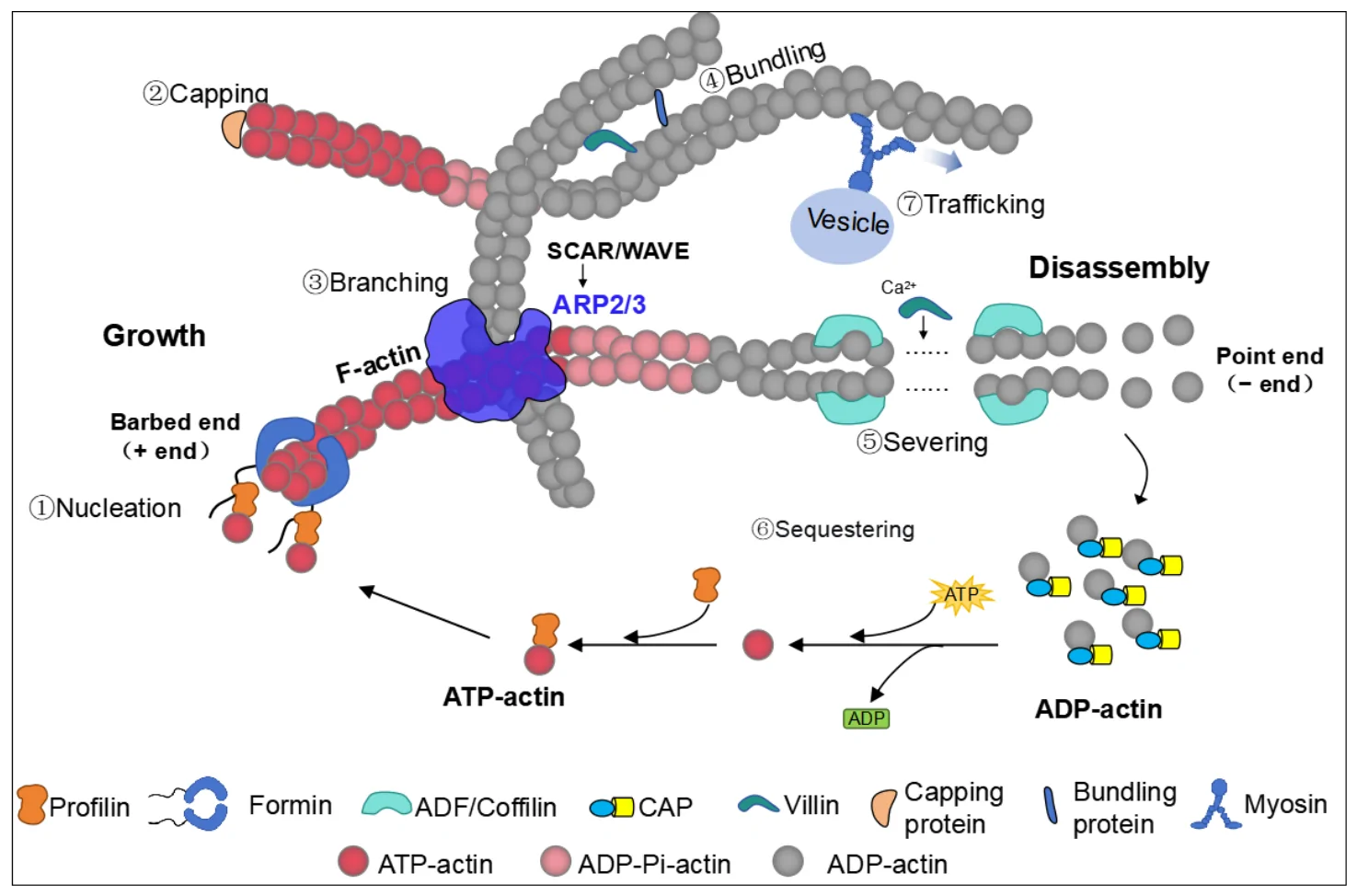
Schematic overview of plant actin cytoskeleton dynamics and the key regulatory actin-binding proteins. Actin cytoskeleton undergoes continuous cycles of filament assembly, remodeling, and disassembly driven by ATP-actin hydrolysis. ① Nucleation and processive growth: Formin binds to the barbed (+) end and processively promotes filament elongation; profilin, by binding ATP-G-actin and interacting with the formin’s proline-rich domains, acts as a chaperone that efficiently transfers the monomer to the formin-associated barbed end for rapid polymerization. ② Capping: Capping protein terminates filament elongation by binding to the barbed (+) end with high affinity, physically blocking the addition of further G-actin monomers. This capping event does not promote disassembly by itself, but it effectively “freezes” the fast-growing end, preventing uncontrolled elongation and limiting the overall filament length. ③ Branching: The ARP2/3 complex, activated by SCAR/WAVE, binds to the side of a pre-existing “mother” actin filament and initiates the de novo growth of a new “daughter” filament at a characteristic 70° angle, generating a dendritic (tree-like) actin network. ④ Bundling: Bundling proteins cross-link adjacent actin filaments in a parallel and closely spaced arrangement to form higher-order structures known as actin bundles. These cross-linkers typically possess two or more actin-binding domains that allow them to simultaneously tether two or more neighboring filaments together. Villin bundles actin filaments under low Ca^2+^ conditions. ⑤ Severing: ADF/Cofilin proteins are the primary severing factors that bind preferentially to the older, ADP-richer regions of actin filaments, distinguishing them from newly polymerized ATP-actin or ADP-Pi-actin segments. Upon binding, ADF/cofilin induces a torsional twist in the filament structure, changing the helical pitch by approximately 5°, which generates mechanical strain at the boundaries between ADF/cofilin-decorated and naked filament regions. This strain leads to a severing event that breaks the filament into shorter fragments, thereby creating new barbed (+) ends and pointed (−) ends. Villin also participates in filament severing, although its severing activity is strictly Ca^2+^-dependent and switches on only upon Ca^2+^ elevation. ⑥ Sequestering (nucleotide exchange and monomer recycling): CAP (cyclase-associated protein) binds ADP-G-actin and promotes nucleotide exchange to regenerate ATP-G-actin; Profilin then binds the ATP-G-actin and delivers it to the barbed end for new rounds of filament polymerization. Actin monomers cycle between ATP-actin, ADP-Pi-actin, and ADP-actin states coupled with ATP hydrolysis and phosphate release. ⑦ Trafficking: Myosin motor proteins associate with actin filaments to drive vesicle trafficking. The pointed (−) end undergoes disassembly, releasing ADP-actin monomers back into the cytoplasmic monomer pool.

AFs are structurally and functionally polarized polymers, a property that arises from the uniform head-to-tail arrangement of asymmetrical G-actin subunits along the filament axis [42] (Figure 1). Each filament possesses two structurally distinct ends, the rapidly growing barbed end (plus end) and the slowly growing pointed end (minus end). The kinetic consequences of filament polarity are profound. Actin polymerization is an ATP-dependent process in which ATP-bound monomers preferentially add to the barbed end at a rate approximately 10- to 100-fold higher than to the pointed end [43]. Following incorporation, ATP is hydrolyzed to ADP, and the inorganic phosphate (Pi) is slowly released, rendering the older regions of the filament enriched in ADP-actin. Under steady-state conditions, this differential rate of subunit addition combined with ATP hydrolysis results in a phenomenon known as treadmilling, in which ATP-actin monomers continuously incorporate at the barbed end while ADP-actin subunits dissociate from the pointed end, maintaining a constant filament length while achieving rapid turnover of subunits [44]. This flux of subunits through the filament is particularly critical in cells requiring rapid cytoskeletal remodeling, such as during cell migration, tip growth, and organelle movement.

The dynamic nature of actin filaments is governed by the intrinsic properties of actin polymerization, such as nucleation, capping, branching, bundling, and disassembly; each tightly regulated by a suite of ABPs (Figure 1). Formin proteins mediate the assembly of unbranched linear actin filaments. They nucleate actin polymerization in cooperation with profilin and remain processively bound to growing barbed ends. At these ends, profilin functions as an actin monomer delivery factor that speeds up elongation by supplying ATP-actin to the formin-associated filament termini [45]. On the other hand, filament nucleation is primarily catalyzed by the ARP2/3 complex, unique in its capability to promote branched filament formation by nucleating daughter filaments from the sides of existing ones [46,47]. Conversely, ADFs (also known as cofilins in animals) promote severing and depolymerization of older or ADP-rich filaments, thereby facilitating rapid filament turnover and creating a pool of monomeric actin for new assembly [48]. To maintain filament stability and higher-order architecture, bundling proteins such as fimbrins and villins cross-link actin filaments into tight parallel bundles or looser networks, which are essential for sustaining cytoplasmic streaming and cortical rigidity under osmotic challenge [49,50,51]. Meanwhile, capping proteins bind to the fast-growing barbed ends of filaments, terminating elongation and preventing uncontrolled polymerization, thus ensuring precise spatiotemporal control of filament length [40,52]. The coordinated actions of these ABPs, rather than any single factor in isolation, determine the cell’s actin reorganization trajectory during diverse cellular processes from vesicle trafficking to polarized cell growth in plants.

### 3.2. The ARP2/3 Complex and WAVE/SCAR Regulatory Network

Among the three major classes of actin nucleators, the ARP2/3 complex holds a central position in plant cells. In its resting state, the complex adopts an autoinhibited conformation in which the two actin-related subunits, ARP2 and ARP3, are spatially separated, rendering the complex intrinsically inefficient as a nucleator [46]. Activation of the ARP2/3 complex is achieved through interaction with nucleation-promoting factors (NPFs), which induce a dramatic conformational rearrangement that brings ARP2 and ARP3 into close proximity, creating a stable dimer that mimics the barbed end of an actin filament [53]. This activated complex then binds to the side of a pre-existing mother filament and initiates the growth of a new daughter filament at a characteristic 70° angle relative to the mother filament [46]. In plants, the ARP2/3 complex is essential for diverse cellular processes, including trichome morphogenesis, pavement cell development, and root hair elongation [54,55,56].

Unlike in animals, where multiple classes of NPFs operate, the SCAR/WAVE (Suppressor of cAMP Receptor/WASP-family Verprolin-homologous) complex serves as the primary activator of ARP2/3 in plants, whereas canonical WASP appears to be absent from land plant genomes [40]. SCAR/WAVE is a conserved pentameric complex composed of SRA1/PIR121 (SCAR-associated protein 1), Nap1 (NCK-associated protein 1), Abi-1/Abi-2 (Abl-interactor 1/2), BRICK1 (BRK1), and the SCAR/WAVE subunit itself [57,58]. Among these subunits, BRK1 plays a particularly critical role, as it selectively stabilizes the SCAR2 subunit and is required for the integrity of the WAVE-ARP2/3 pathway [55]. Loss of BRK1 function severely impairs ARP2/3-mediated actin branching and leads to pleiotropic developmental defects [59].

### 3.3. Actin Motor Proteins: Myosin Family

Beyond structural and regulatory ABPs, actin filaments serve as tracks for molecular motors of the myosin superfamily. In plants, the myosin XI class is the primary motor protein responsible for organelle transport and cytoplasmic streaming [60]. Myosin XI motors move along actin filaments toward the barbed (plus) end, driving the movement of various cargos, including Golgi stacks, peroxisomes, mitochondria, and the endoplasmic reticulum (ER) [61,62].

Two plant-specific myosin subfamilies, myosin VIII and myosin XI, power cytoplasmic streaming, modulate actin architecture, and control cell expansion [63]. In maize and watercress root tissues, the four *Arabidopsis* myosin VIII paralogs accumulate at plasmodesmata (PD) and nascent cell plates, implying a potential role in intercellular transport across PD channels [64,65]. Biochemical evidence shows that myosin VIII can produce or mechanically perceive tensile force. Such mechanical tension optimizes plasmodesmatal transport efficiency and promotes the formation of membrane contact sites linking the endoplasmic reticulum (ER) and the plasma membrane (PM) [66,67].

*Arabidopsis* encodes thirteen myosin XI isoforms. Among them, myosin XI1, XI2 and XIK produce mechanical force to drive the bending and straightening of actin filaments and bundled structures, and accelerate actin cytoskeleton remodeling [68]. In addition, myosin XI participates in vesicular exocytosis and cellulose biosynthesis at the interface connecting the actin cytoskeleton, plasma membrane and cell wall [69]. In particular, myosin XIK is recruited to secretory vesicles prior to the exocyst complex; it presumably recruits and anchors the exocyst to PM docking sites to promote vesicle tethering [70]. Unlike those myosin variants primarily responsible for organelle motility, myosin XIG modulates the dynamics of the F-actin network via independent motor activities [71].

Due to their four conserved functional domains, myosins translocate along actin filaments by hydrolyzing ATP, thereby hauling organelles and macromolecular complexes across the cytoplasm. The tight linkage between myosin activity and cytoplasmic streaming is essential for polar cell growth, which directs directional tip extension in elongated cells including root hairs, pollen tubes and moss protonemata [72,73,74]. Importantly, myosin-mediated cell division operates through mechanisms separate from cytoplasmic streaming [75]. Given that organelle trafficking and cytoplasmic flow persist in both actively growing and mature differentiated cells, future work is required to clarify how motor protein-dependent transport is regulated and coordinated with other cellular processes that maintain intracellular homeostasis [76].

In summary, plant actin filaments are highly polar, dynamically turning-over polymers whose architecture is shaped by a coordinated suite of regulatory factors. Formins and the ARP2/3-SCAR/WAVE complex initiate linear and branched actin networks, whereas severing, capping and bundling ABPs further govern filament stability, geometry and subunit turnover. Plant-specific myosin VIII and myosin XI motors generate mechanical force to support organelle translocation, cytoplasmic streaming and vesicle trafficking, and also participate in reshaping actin arrays. Together, these components constitute the core molecular machinery that enables rapid cytoskeletal rearrangement, which is a prerequisite for stress-responsive actin remodeling. With these basal mechanisms established, we next address how salt stress disturbs actin homeostasis and triggers actin remodeling as part of plant salt-tolerance programmes.

## 4. Dynamic Remodeling of the Actin Cytoskeleton Under Salt Stress

The elaborate actin cytoskeleton described above is not a static architectural scaffold but a highly dynamic, responsive system that continually adjusts its organization in response to environmental cues. As sessile organisms, plants are constantly challenged by adverse environmental conditions, among which salt stress represents one of the most severe constraints on plant growth and productivity worldwide [77]. Upon salt exposure, plant cells experience both osmotic and ionic stresses that trigger a cascade of signaling events, ultimately leading to profound rearrangements of the actin cytoskeleton [48]. These salt-induced actin remodeling events are far from uniform; rather, they exhibit remarkable spatiotemporal specificity depending on cell type, stress duration, and severity. In roots, which serve as the primary site of salt perception and injury, F-actin undergoes characteristic reorganization patterns that range from transient depolymerization and bundling to the formation of specialized arrays, all of which are intimately linked to the regulation of cellular ion homeostasis, cell viability and root growth under saline conditions [78].

In the following section, we summarize current understanding of the remodeling patterns of AFs in response to salt stress, with particular emphasis on the distinct spatiotemporal dynamics of actin reorganization and the regulatory roles of key actin-binding proteins, especially ADFs and bundling proteins, in mediating salt-induced actin rearrangements for salt tolerance and root developmental plasticity.

### 4.1. Spatiotemporal Reorganization of AF Architecture Under Salt Stress

**Figure 2:**
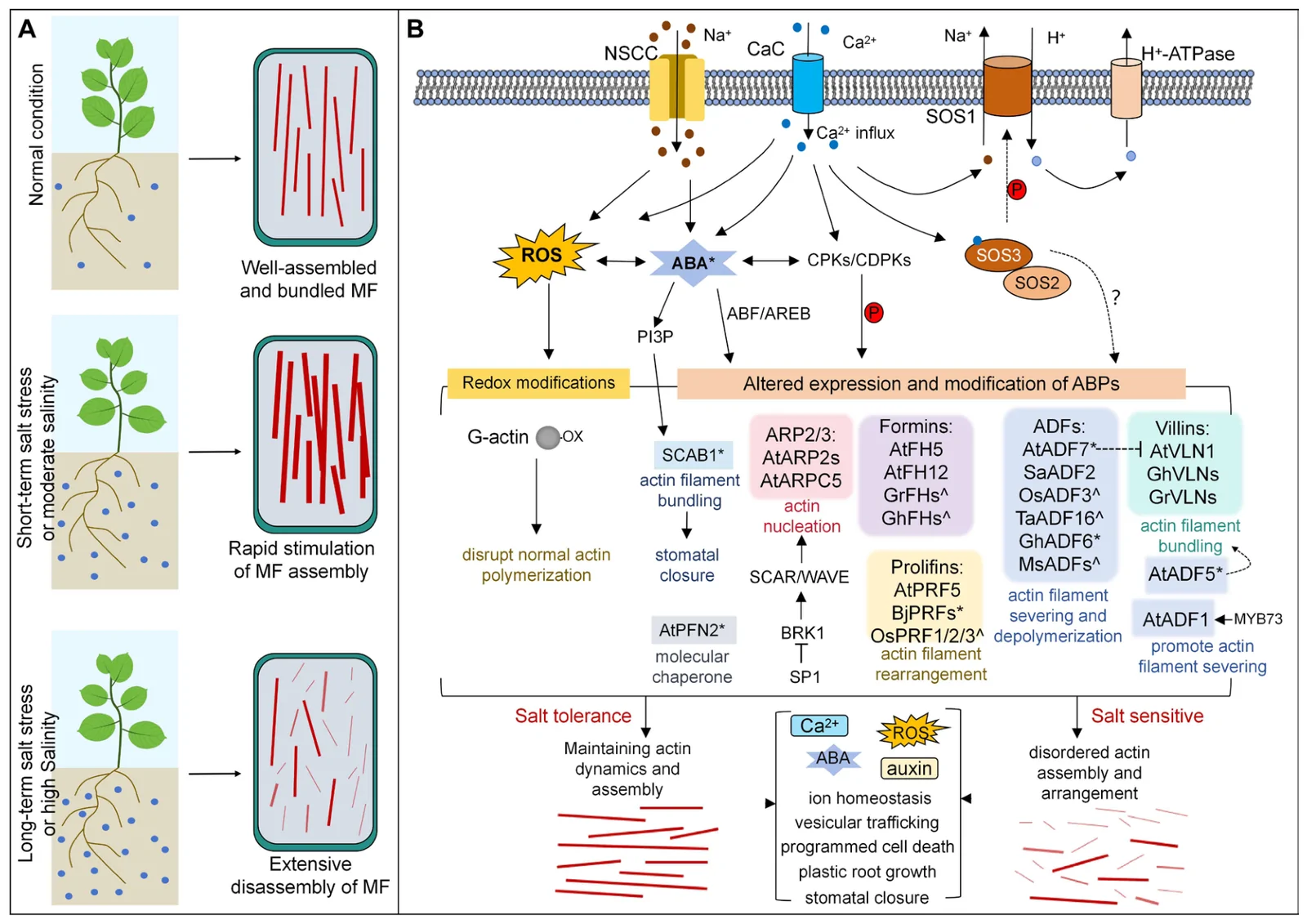
Schematic model illustrating actin cytoskeleton-mediated plant salt-stress response. (**A**) Remodeling of AF arrays under salt stress, based on observations in *Arabidopsis* cotyledon pavement cells [79]. Under normal growth conditions, plant cells exhibit well-assembled and bundled AFs. Upon moderate salinity (150 mM NaCl), AF assembly and bundling are maintained and progressively enhanced over 18 h. Under high salinity (250 mM NaCl), AF fluorescence initially increases before 12 h but declines sharply by 18 h, indicating extensive filament disassembly. Thus, the balance between actin assembly and disassembly is governed by both salt concentration (dose effect) and duration of exposure (time effect), with moderate salinity favouring assembly and prolonged high salinity leading to disassembly. Whether this dose- and time-dependent pattern applies to other tissues and species remains to be determined. (**B**) Working model for the regulatory cascade linking salt-stress signaling, actin-binding protein (ABP) modulation, and actin cytoskeleton rearrangement during plant salt tolerance. Salt stress triggers ion-channel-mediated Na^+^ influx via NSCC and Ca^2+^ entry through CaC channels. Elevated cytosolic Na^+^ activates the SOS salt-overly sensitive pathway (SOS1-SOS2-SOS3 module) for Na^+^ extrusion, while H^+^-ATPase maintains the plasma membrane H^+^ gradient. The salt-induced ROS production thus exerts dual effects: at moderate levels, it promotes actin turnover and stress signaling; at excessive levels, it may cause aberrant polymerization. Elevated Ca^2+^ activates CPK/CDPK kinases, which further modulate ABA signaling. The elevated Ca^2+^ and ABA signaling trigger ROS production, PI3P signaling, and downstream ABF/AREB-dependent transcriptional responses, collectively altering the expression and post-translational modification of diverse ABPs. ABPs include SCAB1 for actin bundling, ARP2/3 complex subunits for actin nucleation, formins (AtFH5, AtFH8, GrFHs, GhFHs), profilin isoforms (AtPRF5, BjPRFs), multiple ADF/cofilin family proteins (AtADF7, SaADF2, OsADF3, TaADF16, GhADF6, MsADFs) that mediate actin filament severing and depolymerization, AtADF5 as a bundling protein supported by ABA/drought evidence, and villin family members (AtVLN1, GhVLNs, GtVLNs) with diverse activities including bundling, Ca^2+^-dependent severing, and capping. Notably, AtADF1 is transcriptionally repressed by MYB73 under normal conditions and facilitates actin-filament severing upon stress-induced derepression of *MYB73*. Meanwhile, AtADF7, which is transcriptionally induced upon salt/osmotic stress, acts as an upstream negative regulator of AtVLN1 by suppressing both its transcript level and its bundling function. Thus, different ADF isoforms are deployed through distinct regulatory mechanisms to remodel actin dynamics under salt stress. The SPI-BRK1-SCAR/WAVE signaling module participates in ARP2/3-dependent actin nucleation. Proper actin dynamics support salt-tolerance-associated outputs, including ion homeostasis, vesicular trafficking, programmed cell death, plastic root growth, and stomatal closure. By contrast, dysregulated actin assembly and arrangement result in salt-sensitive phenotypes. The actin dynamics under salt stress may further feedback to Ca^2+^, ABA, auxin signaling, and ROS accumulation. A question mark denotes an uncharacterized regulatory link in the SOS-ABP signaling axis. *, inferred evidence obtained from non-salt stress (osmotic, drought, cold and heat) or ABA treatment; ^, direct evidence from salt stress with only expression data. Dashed lines indicate unknown regulatory pathways or results derived from non-salt stress studies.

The actin cytoskeleton exhibits rapid and dynamic reorganization in response to salt stress, with the specific remodeling pattern largely determined by stress severity, duration, and cell type (Figure 2A). Using GFP-ABD2 (actin-binding domain 2) as an actin marker, previous research demonstrated that AF organization differs drastically under moderate and high salinity in *Arabidopsis* cotyledon pavement cells [79]. Under 150 mM NaCl, AFs remained well-assembled and bundled, and the fluorescence intensity of GFP-ABD2-labeled AF continuously increased during the first 18 h. In contrast, under 250 mM NaCl, AF fluorescence rose briefly before 12 h and then declined sharply at 18 h, indicating that high salinity triggers transient polymerization followed by extensive disassembly. Thus, moderate salinity favours actin assembly and bundling, whereas prolonged exposure to high salinity drives depolymerization, with the two effects being separable in time. Pharmacological experiments have provided functional evidence for the adaptive significance of this response. Pretreatment with phalloidin, an F-actin stabilizer, significantly enhances plant survival under 150 mM NaCl, whereas treatment with latrunculin A (LatA) and cytochalasin D, which sequesters G-actin and disrupts AFs, drastically reduces seedling viability [79]. It should be noted that this dose- and time-dependent pattern was systematically documented in *Arabidopsis* cotyledon pavement cells; whether the same threshold behaviour applies to other cell types and species requires further investigation.

Rapid F-actin reorganization has also been reported in root cells. Using GFP-ABD2 as an actin marker for live-cell imaging, studies have shown that treatment with 200 mM NaCl disrupts actin filament dynamics within 10 min in the elongation zone of the *Arabidopsis* root tip, manifested as both the fragmentation of long actin bundles and the failure of short actin fragments to elongate into longer filaments [80]. This observation represents a direct measurement of filament dynamics, rather than an inference from fluorescence intensity alone. The spatiotemporal specificity of actin remodeling is further highlighted by studies on gravitropic responses. In live roots expressing GFP-fABD2, quantitative analysis of filament orientation revealed that under 100 mM NaCl treatment, AF orientations in the root elongation zone rapidly shift from an initial axial orientation to a broader distribution with peaks at 15°, 45°, and 90° within just 10 min of exposure [81]. The same study also validated these observations using rhodamine-phalloidin staining of fixed roots. This rapid reorganization contrasts with gravity-induced actin rearrangements, which are slower but result in a higher proportion of microfilaments oriented parallel to the gravity vector. This finding indicates that salt stress triggers a distinct, rapid actin remodeling pattern that may be associated with regulating basic cell extension mechanisms.

Root hairs are another cell type in which salt-induced actin remodeling has been extensively characterized. SPI, a vesicle trafficking protein, stabilizes BRK1 and thereby indirectly regulates actin dynamics in root hairs under salt stress [20]. Live-cell imaging of root hairs of the ABD2-GFP marker line has revealed that salt stress induces altered actin organization in root hairs that phenocopies the actin defects observed in *spi* mutants [20]. As SPI modulates BRK1 protein stability, loss of SPI function leads to the accumulation of steady-state BRK1 protein, resulting in aberrant actin organization and severely impaired root hair development under salt stress. Consistent with a role for actin dynamics in stress adaptation, overexpression of *BRK1* causes root hair hypersensitivity to salt stress [20]. Thus, while the SPI-BRK1 study provides direct evidence linking a trafficking protein to actin organization in root hairs, its conclusions are based on visual assessment of filament organization rather than quantitative measurements of filament turnover, leaving the precise dynamics of actin remodeling in this system to be further resolved.

Progressive filament fragmentation and massive actin depolymerization under severe salt stress may correlate closely with elevated rates of programmed cell death (PCD). It was demonstrated that salt-induced ion disequilibrium triggers PCD in both yeast and plants [82]. The study showed that NaCl induces the cytological hallmarks of lysigenous-type PCD, including nuclear fragmentation, vacuolation, and lysis, and that salt-sensitive mutants of yeast (*cnb1Δ*) and *Arabidopsis* (*sos1*) exhibit substantially more profound PCD symptoms, confirming that salt-induced PCD is mediated by ion disequilibrium [82]. A commentary noted that actin depolymerization represents an evolutionarily conserved core event in PCD [83]. This view is consistent with broader evidence that the actin cytoskeleton plays an active, functional role in determining whether PCD is triggered and executed across yeast, plants, and animals [84]. Together, these observations suggest a conserved link between actin depolymerization and PCD under salt stress, although direct manipulation of actin dynamics within the same experimental system will be required to establish a causal relationship.

### 4.2. Regulatory Roles of ABPs in Salt-Induced Actin Remodeling

Fine-tuning of actin nucleation, filament severing, bundle assembly and actin monomer recycling under salinity is jointly orchestrated by nearly all core families of ABPs. Below, we systematically summarize these salt-responsive ABPs together with the corresponding genetic evidence, as summarized in Table 1. A working model of the regulatory cascade linking ABP modulation, salt-stress signaling, and actin cytoskeleton rearrangement during plant salt tolerance is illustrated in Figure 2B.

#### 4.2.1. ADFs as Central Modulators of Salt-Induced Actin Turnover

Among the ABPs that respond to salt stress, ADFs are the most extensively characterized family, functioning as central regulators of F-actin turnover during salt exposure [48,105]. ADFs exhibit context-dependent activities that vary according to cell type, stress severity, and developmental context. Collectively, they orchestrate a precise balance between AF severing/depolymerization and filament stabilization to support salt adaptation.

In *Arabidopsis*, *AtADF1* is rapidly upregulated at the transcriptional level upon salt exposure. Under normal conditions, the MYB73 transcription factor directly binds to the ADF1 promoter and represses its expression. However, salt stress suppresses MYB73, thereby releasing ADF1 from transcriptional inhibition [85]. Functional analyses have demonstrated that the *adf1* null mutant accumulates abnormally thick actin bundles, fails to reorganize AF in response to NaCl, and exhibits drastically reduced survival rates under salinity [85]. Mechanistically, AtADF1 severs excess actin cables to maintain the dynamic filament equilibrium that is essential for the cycling of Na^+^ transporters at the plasma membrane, thereby preserving ion homeostasis [85]. This dual role, both depolymerizing and severing, positions AtADF1 as a critical homeostatic regulator that prevents pathological actin bundling while sustaining functional transporter dynamics.

The functional diversification of ADF family members is further illustrated by ADF5 and ADF7, which operate under distinct cellular contexts. AtADF5 stabilizes F-actin bundles under drought stress to support stomatal closure, a process that requires precise actin reorganization in guard cells [86]. Mechanistically, the ABA-responsive transcription factor DPBF3 directly binds the ADF5 promoter to activate its expression, linking ABA signaling to ADF5-mediated actin bundling during stomatal closure. Another example of functional specialization is AtADF7, whose antagonistic interplay with the actin-bundling protein VLN1 fine-tunes F-actin architecture in root hairs under osmotic stress [78], which is discussed in detail in Section 4.2.3. Although these studies primarily address drought and osmotic stress responses, similar regulatory mechanisms presumably operate under saline conditions. However, the functional divergence between ADF5 (bundling in guard cells) and ADF7 (severing in root hairs) raises an important unresolved question: is ADF isoform specificity determined primarily by cell type or by stress type? ADF5 bundles under drought, ADF7 severs under osmotic stress, and ADF1 severs under salt, yet all three are ADF family members with conserved actin-binding domains. This suggests that isoform-specific functions may arise less from intrinsic biochemical differences than from spatiotemporal expression and interaction partners. Testing this hypothesis would require swapping ADF coding sequences while retaining native promoters, an experiment that has not yet been performed.

Homologs of salt-responsive ADF proteins have been discovered across diverse plant species, pointing to an evolutionarily conserved regulatory strategy in the plant kingdom. In rice, *OsADF3* is transcriptionally activated by salinity and drought. Heterologous overexpression of this gene in *Arabidopsis* improves stress resistance, as reflected by higher germination ratios, longer primary roots and elevated survival rates. This protective effect is accompanied by elevated transcript levels of stress marker genes including *RD22*, *ABF4*, *DREB2A* and *RD29A* [87]. In wheat (*Triticum aestivum*), *TaADF16* has been characterized as a positive regulator of cold tolerance [88], while its expression is also induced by salt stress, albeit to a lesser extent than by cold [89]. In cotton (*Gossypium hirsutum*), GhADF6 participates in salt stress adaptation, and its suppression under alkaline stress is correlated with AF stabilization [90]. More recently, a systematic genome-wide analysis of the *ADF* gene family in alfalfa *(Medicago sativa*) identified nine *MsADF* genes, the majority of which were induced by salt and drought stress by more than two-fold, with MsADF1, MsADF2/3, MsADF6, and MsADF9 being highly induced [91], suggesting their critical roles in abiotic stress resistance. While the biological functions of most of these ADF members remain uncharacterized, their involvement in salt stress responses is likely linked to the dynamic stability of AFs.

The adaptive significance of ADF-mediated actin regulation is underscored by studies on halophytes. Heterologous overexpression of *SaADF2*, a constitutively upregulated *ADF* from the halophyte *Spartina alterniflora*, in rice significantly improves salt resistance by sustaining intact actin architecture and preserving the photosynthetic apparatus [92]. This gain-of-function approach highlights the potential of ADF engineering for enhancing crop salinity tolerance and validates the concept that optimized actin dynamics confer stress resilience.

A critical assessment of this body of evidence, however, reveals a substantial gap between expression-level observations and functional validation. Among the ADF homologs surveyed above, only AtADF1, OsADF3 and SaADF2 have been functionally validated through loss-of-function or gain-of-function experiments; the majority, including the wheat, cotton and alfalfa ADFs, have been characterized only at the transcript level. Moreover, the direction of regulation is not consistent across species: GhADF6 is downregulated under alkaline stress, whereas most MsADFs are upregulated under salt, and TaADF16 responds far more strongly to cold than to salt. These inconsistencies suggest that ADF-mediated salt responses may be species-specific and stress-type-specific, and that findings from one crop cannot be readily extrapolated to another. A systematic, cross-species comparison of ADF loss-of-function and gain-of-function lines under standardized salt conditions would therefore be a valuable community resource, and would help distinguish conserved core functions from lineage-specific adaptations.

#### 4.2.2. The ARP2/3 Complex in Salt-Triggered Branched Actin Nucleation

Emerging evidence has positioned the ARP2/3 complex as a key player in plant responses to salt stress. The mechanistic basis for this regulation involves a mitochondria-associated Ca^2+^ signaling pathway. Genetic screening of *Arabidopsis* mutants defective in stress-induced increases in cytosolic Ca^2+^ identified ARP2 as a critical regulator of salt-induced Ca^2+^ signaling [93]. Salt-exposed plants lacking *ARP2* or other subunits (*arp2-1*, *arp2-2*, and *arpc5*) of the ARP2/3 complex exhibited a loss of organized actin structures, resulting in accelerated F-actin breakdown, mislocalization of actin filaments and increased sensitivity to salt stress. This actin disorganization is accompanied by enhanced increases in cytosolic Ca^2+^, mitochondrial aggregation, increased permeability transition pore, and impairment of mitochondrial membrane potential. These mitochondrial dysfunctions were closely associated with salt-induced PCD. When transferred to medium containing 200 mM NaCl, these mutants showed significantly reduced survival rates compared to wild-type plants, whereas complementation lines largely restored salt tolerance [93].

The upstream regulatory network of the ARP2/3 complex, especially SCAR/WAVE complex, is also relevant to salt stress adaptation. Recent studies have identified SPI, a BEACH domain-containing protein, as an upstream regulator of BRK1 stability [20]. SPI negatively regulates BRK1 protein accumulation. Functional loss of SPI results in the accumulation of steady-state BRK1, consequently impairing SCAR/WAVE complex assembly and ARP2/3-mediated actin nucleation, leading to aberrant actin organization and root hairs that are hypersensitive to salt stress [20]. Consistently, overexpression of BRK1 caused root hairs to become over-sensitive to salt stress, indicating that precise regulation of BRK1 levels and, thereby, ARP2/3-mediated actin nucleation, is crucial for salt-induced root hair developmental plasticity. This SPI–BRK1–SCAR/WAVE regulatory axis thus links a BEACH-domain protein to the conserved actin-nucleating machinery and emerges as a key determinant of root hair developmental plasticity in response to salt stress [20].

Together, these two lines of evidence position ARP2/3 not merely as an actin nucleator but as a convergence point for Ca^2+^-mitochondrial signaling and developmental plasticity under salt stress. Whether these two regulatory inputs act independently or converge on a common ARP2/3 activation step requires further investigation.

#### 4.2.3. Villins as Dual-Function Bundling/Severing Proteins Under Saline Conditions

Villins (VLNs) are a conserved family of actin-binding proteins that belong to the villin/gelsolin/fragmin superfamily, characterized by the presence of six gelsolin homology domains (G1–G6) and an additional villin headpiece domain (VHP) at the C-terminus [106]. In plants, villins function as multifunctional actin regulators capable of nucleating, capping, severing, and bundling actin filaments, making them critical modulators of actin cytoskeleton dynamics under both normal and stress conditions [50,95].

The *Arabidopsis* genome encodes five villins (AtVLN1–AtVLN5), which are classified into three phylogenetic groups with distinct biochemical properties [107,108]. Group I member AtVLN1 crosslinks and stabilizes actin filaments but lacks actin nucleation, severing, and barbed-end capping capacities [50]. In contrast, the remaining four AtVLNs are “all-rounders” that participate in actin nucleation, bundling, severing, and capping [95]. Such broad functional divergence enables villins to precisely calibrate actin cytoskeleton architecture to accommodate diverse cellular requirements. Biochemical and genetic evidence indicates that AtVLN1 assembles stable actin filament cables that resist depolymerization, shielding F-actin from ADF-driven disassembly [50]. This actin-stabilizing function becomes physiologically vital during salt stress, as intact actin cytoskeletal structures are indispensable to sustain cell viability under saline conditions [78,94]. As introduced in Section 4.2.1, the antagonistic AtADF7-AtVLN1 relationship provides a clear example of how ADF-mediated severing and VLN1-dependent bundling counterbalance each other to drive adaptive actin remodeling in root hairs [78]. This equilibrium drives the adaptive actin remodeling essential for proper root hair initiation and elongation in stress environments. However, whether VLN1 acts primarily as a passive shield against depolymerization or as an active organizer that spatially instructs ADF7 activity remains an open question.

Genome-wide analysis of cotton (*Gossypium hirsutum*) identified 14 *GhVLN* genes, many of which were upregulated by salt, drought, cold, and heat stresses [96]. The promoter regions of *GhVLN* genes contain various stress-responsive cis-elements, including those associated with abscisic acid (ABRE), drought (MBS), and defense responses (TC-rich repeats) [96]. Notably, *GhVLN4* was significantly upregulated by NaCl treatment, and its overexpression in *Arabidopsis* enhanced salt tolerance, characterized by upregulated components in both ABA-related and ABA-independent signaling pathways [95]. These findings suggest that villins may contribute to salinity tolerance by modulating stress signaling cascades, in addition to their direct effects on actin cytoskeleton organization. Notably, most evidence linking villins to salt tolerance currently comes from expression profiling or heterologous overexpression, and loss-of-function evidence in the target crop is still largely lacking.

Collectively, villins represent an important but relatively underexplored class of actin-binding proteins in salt-induced actin remodeling. Their ability to both stabilize actin filaments and coordinate with ADF-mediated turnover positions them as key integrators of actin dynamics during salt stress adaptation. Future work should prioritize isoform-specific genetic analysis in crops, since the current reliance on heterologous systems and expression patterns limit conclusions about which villin isoforms are functionally rate-limiting for salt tolerance in the target species.

#### 4.2.4. Profilins as G-Actin Buffers Maintaining Polymerization Competence Under Ionic Stress

Profilins (PRFs) are small actin-binding proteins (12–15 kDa) that play a pivotal role in regulating actin dynamics by controlling the rate of actin polymerization, bundling, and cable formation. Profilin prevents aberrant spontaneous actin nucleation and shuttles charged actin monomers to growing barbed ends to support filament elongation [109]. In animal cells, profilin is responsible for converting ADP-actin monomers to ATP-actin to recharge monomers for actin assembly [110]. Evidence accumulated to date, however, indicates that plant profilin exhibits poor activity in stimulating actin nucleotide exchange. Instead, this essential biochemical function in plants is carried out by cyclase-associated protein 1 (CAP1) [111]. In plants, actin recycling is thus driven by a three-component cooperative cycle. ADF/cofilin severs and depolymerizes aged ADP-actin filaments to release ADP-actin monomers. CAP1 acts as the nucleotide-exchange factor to convert ADP-actin into ATP-actin, a biochemical function that plant profilin is unable to execute. Profilin then binds ATP-actin and shuttles these monomers to filament barbed ends to support reassembly. Together with ADF/cofilin and CAP1, profilin sustains continuous monomer cycling between filament disassembly and new assembly, thereby maintaining steady-state actin turnover and cytoskeletal remodeling [111].

In plants, PRFs are encoded by multigene families. For instance, *Arabidopsis* contains five *PRF* genes [112], whereas rice harbors three members (*OsPRF1*-*3*) [97]. Segmental and tandem duplications have shaped functional diversification among PRF isoforms, with purifying selection maintaining their core actin-binding functions while leaving room for adaptive diversification in stress-responsive regulation [113]. Accumulating evidence supports the involvement of profilins in salt and osmotic stress responses. Transcriptional profiling in rice revealed differential expression of *PRF* genes under abiotic stress and hormone treatments, pointing to their roles in distinct stress signaling pathways [97]. Similarly, *Brassica juncea BjPRF* members display divergent expression patterns across different stress conditions [98].

An important mechanistic discovery confirmed that osmotic stress triggers profilin-dependent actin reorganization to sustain cellular and tissue integrity. Using transcriptomics, phosphoproteomics and forward genetics, researchers screened *Chlamydomonas reinhardtii* for osmotic tolerance and identified 76 genes, mainly involved in actin cytoskeleton organization, ion transport, vesicle trafficking, MAPK cascades and chloroplast function [99]. Comparative analysis showed that the algal osmotic stress network shares 24% overlap with *Arabidopsis*, far exceeding the 6% similarity with yeast, highlighting the evolutionary conservation among green plants. This work pinpointed profilin-mediated actin remodeling as a core mechanism for maintaining tissue integrity under osmotic challenges. Moreover, it validated PRF5 as a central regulator, as *Arabidopsis prf5* mutants were hypersensitive to NaCl and mannitol, accompanied by disordered actin architecture and reduced cell viability under stress [99].

Phenotypic characterization of *prf5* mutant roots further elucidates how PRF5 governs stress-induced actin rearrangement. In wild-type seedlings, osmotic stress reorganizes actin filaments into longitudinal cables parallel to the cell elongation axis, which stabilizes cell walls and mitigates plasmolysis [99]. By contrast, salt treatment triggered premature and excessive actin bundling in *prf5* roots. This defect caused exaggerated calcium oscillations and premature cell death in the elongation zone. Even mild osmotic shock (250 mM mannitol) induced severe plasmolysis in mutant roots, whereas wild-type cells remained unaffected [99]. Treatment with the actin-depolymerizing drug Latrunculin B (LatB) recapitulated the mutant phenotype, verifying that PRF5-mediated actin dynamics are essential for osmotic resistance. These results support a working model, in which PRF5 delays osmotic stress-induced actin bundling, enabling gradual cellular acclimation and preventing destructive cytoskeletal rearrangement that leads to cell death. The elevated frequency of calcium spikes observed in stressed *prf5* mutants provides additional evidence for this model [99]. However, whether this “delay” function reflects a direct inhibitory effect of PRF5 on bundling proteins or an indirect consequence of PRF5-mediated maintenance of G-actin pools remains to be determined.

Beyond their canonical role in actin dynamics, certain plant profilin isoforms exhibit molecular chaperone activity that may contribute to stress tolerance. In *Arabidopsis*, AtPFN2, which exists predominantly as high molecular weight (HMW) homo-oligomeric complexes (ranging from ~130 to >440 kDa), has been demonstrated to function as a holdase chaperone [100]. This chaperone activity was evidenced by the ability of AtPFN2 to prevent the heat-induced aggregation of the model substrate malate dehydrogenase (MDH) in a dose-dependent manner under heat shock conditions (43 °C), whereas AtPFN1, which adopts low molecular weight (LMW) forms, lacked such protective capacity [100]. Although this chaperone activity has been characterized primarily under heat shock conditions, it suggests that profilin isoforms may play a broader role in protecting cellular proteins from denaturation under various abiotic stresses. This chaperone function could indirectly contribute to the maintenance of actin monomer integrity and overall cellular proteostasis under stress conditions, though direct evidence for actin monomer protection specifically under salt-induced oxidative stress remains to be established.

#### 4.2.5. Formins as Linear Actin Nucleators Directing Polarized Growth During Salt Adaptation

Formins constitute a large, evolutionarily conserved family of actin-nucleating proteins characterized by the tandem presence of Formin Homology 1 (FH1) and Formin Homology 2 (FH2) domains [114]. The FH2 domain dimerizes to nucleate actin filament assembly and processively caps the growing barbed end, while the FH1 domain, consisting of polyproline tracts, recruits profilin–actin complexes and delivers actin subunits to the elongating filament end [114,115,116]. This unique “processive capping” mechanism allows formins to catalyze linear actin polymerization without requiring a pre-existing filament nucleus, distinguishing them from the ARP2/3 complex that promotes branched actin networks [114].

In angiosperms, formins are divided into two classes based on FH2 domain phylogeny and domain architecture. Class I formins typically possess N-terminal transmembrane domains and extracytoplasmic regions, positioning them as membrane-embedded organizers at the plasma membrane and endomembrane compartments [115]. By nucleating linear actin arrays at the cell periphery, Class I formins establish directional actin cables that support polar tip growth and orient root elongation trajectories. Class II formins, in contrast, feature membrane lipid-binding domains related to PTEN (Phosphatase and Tensin Homolog), enabling peripheral membrane association [115]. Such membrane-targeting properties allow formins to tightly couple polar cell wall growth and actin remodeling with upstream signal transduction [117]. Formin-mediated actin assembly guides cytoskeletal polarization and participates in root hair initiation and tip growth [118], which is essential for steering root directional growth and maintaining robust polar cell expansion when plants confront adverse environmental conditions [119,120].

Multiple lines of evidence have established the functional significance of formins in salinity tolerance. In *Arabidopsis*, the Class II formin AtFH12, whose expression is induced by NaCl, exhibits an actin-associated function revealed through genetic interaction studies. While *atfh12* mutants display negligible phenotypic defects under salt stress alone, the mutation dramatically exacerbates the toxic effects of an actin-disrupting fluorescent marker, suggesting functional redundancy within the formin family that masks individual contributions [101]. The Class I transmembrane formin AtFH5 exhibits salt-inducible expression in seedling root tissues, with transcript levels markedly increased upon NaCl treatment [22]. Notably, *atfh5* loss-of-function mutants display heightened sensitivity to salt stress, whereas introduction of AtFH5-GFP rescues this phenotype. Subcellular localization studies reveal that AtFH5-GFP accumulates in border-like cells of the root cap, phloem tissues, and lateral root emergence sites, regions that represent critical interfaces for ion uptake and transport. Interestingly, protein distribution under salt stress does not fully mirror transcript abundance, suggesting post-transcriptional regulation potentially involving endocytosis and autophagic trafficking [22]. Consistent with this, salt stress induces autophagy in *Arabidopsis*, and AtFH5 may function as an “active cargo” of membrane trafficking, modulating the fate of autophagic compartments en route to vacuolar destinations. The observation that mannitol-induced osmotic stress does not upregulate AtFH5 indicates that this formin responds specifically to ionic stress rather than osmotic perturbation alone. However, the direct experimental evidence regarding its impact on F-actin dynamics under salt stress remains to be established. This gap is notable because AtFH5 is one of the few formins for which salt-specific loss-of-function evidence exists; determining whether its salt-induced expression translates into measurable changes in actin architecture would clarify whether formins contribute to salt tolerance primarily through actin nucleation or through membrane trafficking functions.

Beyond *Arabidopsis*, genome-wide analyses across other species reinforce the connection between formins and salt adaptation. For instance, 26 *FORMIN* genes (*GrFHs*) were identified from the wild diploid cotton, *Gossypium raimondii*, with *GrFH19* and *GrFH26* showing significant upregulation under salt stress [102]. Promoter regions of these genes harbor cis-acting regulatory elements associated with stress responses, and transcription factor families including ERF, GATA, MYB, and LBD are implicated in their salt-responsive regulation [102]. Likewise, 46 *FORMIN* genes (*GhFHs*) were identified from domesticated tetraploid cotton (*Gossypium hirsutum* L.), with *GhFH20* and *GhFH30* exhibiting increased expression under salt treatment and could play significant roles in the development of *G. hirsutum* under heat and salt stresses [103]. Future studies leveraging genome-wide expression profiles and tissue-specific functional analyses across diverse species will further elucidate the regulatory networks governing formin-mediated stress resilience and inform breeding strategies for salinity-tolerant crops.

In summary, salt-induced actin cytoskeleton remodeling displays prominent spatiotemporal heterogeneity, including moderate salinity favouring actin assembly and bundling, whereas severe, long-term salt exposure causes filament depolymerization linked to programmed cell-death risk. A suite of ABPs, covering ADF/cofilin, the ARP2/3-SCAR/WAVE complex, villins, profilins and formins, are transcriptionally or post-translationally adjusted under salt stress. Distinct ABP isoforms often exert opposing or counter-balancing biochemical activities, forming a finely tuned regulatory network governing actin nucleation, severing, bundling and monomer turnover. Genetic studies in model plants, crops and halophytes demonstrate that appropriate actin dynamics support key salt-tolerance outputs including ion homeostasis, plastic root growth and cell viability. Nevertheless, many ABPs remain functionally uncharacterized under salt conditions, and the signalling cascades linking early salt perception to ABP activation are not fully resolved.

## 5. Signaling Cascades Linking Salt Sensing to Actin Remodeling

Salt stress triggers signaling cascades that dynamically reorganize the actin cytoskeleton to support plant adaptation. This section delineates the major upstream signaling modules, including the Ca^2+^-SOS pathway, calcium-dependent protein kinases (CPKs), the ROS-actin feedback loop, and ABA-mediated cross-talk, that transduce salt perception into actin remodeling.

### 5.1. The Ca^2+^-SOS Signaling Module: Primary Salt Signal to Actin

Salt stress elicits a rapid and transient elevation of cytosolic Ca^2+^ concentration, which serves as the primary ionic signal decoded by the SOS pathway—the best-characterized signaling cassette for Na^+^ homeostasis in plants [121]. The SOS pathway comprises three core components: the Ca^2+^ sensor SOS3 (a myristoylated calcium-binding protein), the serine/threonine protein kinase SOS2, and the plasma membrane Na^+^/H^+^ antiporter SOS1 [122]. Upon salt-induced Ca^2+^ elevation, SOS3 binds Ca^2+^, undergoes conformational changes and dimerization, and subsequently interacts with and activates SOS2 via the FISL motif in its regulatory domain. The activated SOS2–SOS3 complex then phosphorylates and stimulates SOS1, promoting Na^+^ extrusion from the cytosol.

Crucially, this canonical ion-homeostasis pathway is directly coupled to actin cytoskeletal dynamics. Phalloidin-facilitated AF polymerization increased survival of the salt-hypersensitive *sos2* mutants and rescued their stress-related defects [79], suggesting that a functional connection exists between AF assembly and SOS2-mediated salt tolerance. However, whether AF assembly acts downstream of or in parallel to SOS2 signaling has not been directly established, as distinguishing these two possibilities requires genetic interrogation (e.g., double-mutant analysis), rather than pharmacological rescue alone. Moreover, the *sos3* loss-of-function mutant exhibits pronounced abnormalities in actin filament organization under salt stress, characterized by disordered actin assembly and arrangement that are more severe than in wild-type plants [123]. Remarkably, the salt hypersensitivity of *sos3* mutants can be partially rescued either by exogenous calcium supplementation or by low concentrations of latA, an actin monomer-sequestering agent, that reduces actin polymerization. These parallel effects of calcium and latA on actin organization and salt sensitivity strongly suggest that the salt hypersensitivity of *sos3* mutants partially stems from their disrupted actin cytoskeleton, positioning SOS3-mediated actin filament reorganization as an integral component of plant salt tolerance. This finding establishes a direct mechanistic link between salt perception (Ca^2+^ signature) through the SOS signaling module and actin remodeling, although the precise molecular target of SOS3/SOS2 within the actin regulatory network, such as whether an actin-binding protein or a direct actin-interacting partner, remains to be fully elucidated.

In plants, investigations into Ca^2+^ signal alterations triggered by actin remodeling are conceptually similar to those in the animal kingdom, and likewise demonstrate that this constitutes a bidirectional and dynamic regulatory loop. Although direct investigations focusing on actin remodeling-mediated calcium signaling under salt stress are still lacking, studies on pollen tube polar growth, plant immune defense and leaf movement have demonstrated that the plant actin cytoskeleton acts as both a “decoder” of calcium signals and, in a reciprocal manner, a “modulator” governing the spatiotemporal dynamics of calcium signaling [124]. At the tip of the pollen tube, there exists a sustained intracellular calcium ion concentration gradient, which serves as the basis for its polar growth. Dynamic changes in actin can regulate calcium-permeable channels on the plasma membrane, thereby influencing the establishment and maintenance of this calcium gradient, and consequently modulating the direction of pollen tube growth [125]. In grapevine cells, chitosan, a fungal cell wall component, can rapidly trigger defense responses, including calcium influx and actin remodeling [126]. Interestingly, this study revealed a sophisticated negative feedback regulatory mechanism: the calcium influx triggered by chitosan suppresses the transcriptional activation of certain defense genes (such as those related to phytoalexin biosynthesis). This suggests that calcium signaling may act as a “brake” to prevent excessive defense responses, ensuring the transience and precision of the signal. Furthermore, the semimonastic (touch-induced) movement of the sensitive plant (*Mimosa pudica*) provides direct evidence that actin dynamic changes lead to alterations in calcium signaling. When subjected to touch or electrical stimulation, the actin filaments within the motor cells of the pulvinus rapidly depolymerize and fragment. Studies have found that this disruption of the actin cytoskeleton is an upstream event that directly leads to a significant elevation in intracellular calcium levels, ultimately triggering water efflux and cell contraction, resulting in leaf closure [127]. When actin is stabilized with drugs such as phalloidin, both the calcium signal elevation and the movement are inhibited, clearly demonstrating the causal relationship between actin remodeling and changes in calcium signaling.

Uncovering how plants distinguish salt stress from other abiotic factors will require examining the subsecond, single-cell dynamics between actin regulators and calcium channels. Advances in live-cell imaging, combined with targeted mutagenesis of actin-binding domains in calcium channels and characterization of additional actin-binding proteins such as villin/gelsolin/fragmin superfamily members and ADF/cofilin, promise to reveal whether actin remodeling operates upstream or in concert with calcium-induced calcium release during salt adaptation. Ultimately, unravelling these actin–calcium regulatory loops may reveal new strategies for engineering salt-tolerant crops by manipulating the very choreography that dictates signal specificity.

### 5.2. Calcium-Dependent Protein Kinases Mediate ABP Phosphorylation

The transient Ca^2+^ signal elicited by salt stress is also decoded by CPKs, a large family of Ser/Thr protein kinases that directly translate Ca^2+^ signals into phosphorylation events [128]. Unlike the SOS pathway, which primarily targets ion transporters, CPKs are strategically positioned to modulate actin-binding proteins through phosphorylation, thereby exerting rapid and reversible control over actin dynamics.

Several CPK isoforms have been implicated in salt stress responses [129]. For instance, in *Arabidopsis*, CPK3 is activated within minutes of NaCl treatment [130]. Phenotypically, *cpk3* loss-of-function mutants exhibit reduced germination rates under salt stress, whereas CPK3-overexpressing lines show improved germination, establishing a positive correlation between CPK3 abundance and salt tolerance during early development [130].

The connection between CPKs and actin dynamics is further supported by studies in the ice plant (*Mesembryanthemum crystallinum*). McCPK1, a salt- and dehydration-inducible CPK, undergoes myristoylation that contributes to its plasma membrane localization [128]. Strikingly, McCPK1 exhibits reversible subcellular redistribution from the plasma membrane to the nucleus, endoplasmic reticulum, and actin filaments of the cytoskeleton in response to low humidity [128]. This dynamic relocalization to the actin cytoskeleton implies that CPKs may directly associate with actin-associated proteins or even actin itself under stress conditions. At present, direct phosphorylation targets of CPKs within the actin regulatory network remain largely uncharacterized under salt stress, although CPK3/CPK6-mediated phosphorylation of SCAB1 (STOMATAL CLOSURE-RELATED ACTIN BINDING PROTEIN1) has been demonstrated under osmotic stress [131] (see Section 5.4 for detailed discussion). Further identification of CPK substrates among core actin-binding proteins, such as ADF/cofilin, profilin, villins and formins, is therefore required to fully dissect the molecular cascade connecting CPK signaling to actin remodeling under salt stress.

### 5.3. ROS-Actin Feedback Loop

ROS act as dual-function signaling molecules and toxic stress byproducts under salinity, drought and irradiation, exerting dose-dependent control over plant actin architecture. Conversely, actin remodeling shapes cellular ROS homeostasis to form a conserved feedback regulatory circuit in eukaryotes [132].

Excess stress-triggered ROS induces redox modifications on actin monomers and ABPs, directly disturbing the balanced turnover of G- and F-actin [133]. Oxidation of cysteine and methionine residues within actin triggers intermolecular disulfide crosslinkages, driving the formation of aberrant actin aggregates termed oxidation-induced actin bodies (OABs), which were first characterized in yeast [134]. Plant actins also form OABs. ROS can directly modify actin monomers through oxidative post-translational modifications. For instance, in *Arabidopsis* cells exposed to 2,4-D-induced oxidative stress, actin undergoes oxidation and S-nitrosylation at specific cysteine residues. These modifications disrupt normal actin polymerization, as evidenced by a decreased F-actin/G-actin ratio, ultimately compromising cytoskeletal integrity and organelle dynamics [135]. In rice root cells, H_2_O_2_ treatment triggers the formation of high-molecular-weight actin aggregates linked by intermolecular disulfide bonds. The cellular chaperone protein disulfide isomerase PDI1;1 recognizes and reduces these aberrant disulfide cross-linkages, facilitating the degradation of oxidized actin aggregates [136]. This mechanism represents a cellular quality control system that mitigates ROS-induced actin damage and maintains cytoskeletal homeostasis under oxidative stress.

Low, physiological ROS signals establish apical gradients in root hairs and pollen tubes, which facilitate ordered actin polymerization to sustain directional tip elongation [137]. ROS-derived signal transduction serves an indispensable regulatory role in remodeling actin architecture during pollen self-incompatibility reactions and ABA-mediated stomatal aperture adjustment [138,139]. The ROS-mediated actin remodeling is further demonstrated to rely on the heterodimeric capping protein (CP) based on a study of flg22-induced immunity. The CP responds and transduces ROS signaling to the actin cytoskeleton, as H_2_O_2_ treatment failed to elicit actin remodeling in *cp* mutants but restored the increase in AF in CP-overexpression cells [140].

Actin remodeling under salt stress also feeds back on ROS homeostasis. In the root elongation zone of *Arabidopsis* seedlings, 200 mM NaCl disturbs actin filament dynamics within 10 min, accompanied by a significant increase in ROS levels [80]. Notably, pharmacologically induced actin depolymerization (LatB) and excessive stabilization (jasplakinolide, Jasp) both intensified ROS accumulation under NaCl treatment, suggesting that disrupted actin dynamics, rather than a specific polymerized or depolymerized state, triggers the ROS response [80]. This result demonstrates that ROS production is provoked by perturbed actin homeostasis, instead of being tied exclusively to either the polymerized or depolymerized state of microfilaments [80]. Furthermore, pharmacological inhibition of NADPH oxidase with diphenyleneiodonium (DPI) abolishes this salt-induced ROS burst. This observation indicates that actin remodeling modulates ROS generation through an AtrbohC-dependent pathway, as demonstrated by the loss of salt-induced ROS accumulation in *atrbohC* mutants [80].

Conversely, ROS can feed back to modulate actin organization. The *rsa3-1* mutant in *Arabidopsis*, which is hypersensitive to salt stress, accumulates excessive ROS under saline conditions [141]. The *RSA3* gene encodes a xyloglucan galactosyltransferase with a dual function. It participates in cell wall biosynthesis and, critically, regulates AF organization [141]. In *rsa3-1* mutants, actin fails to assemble into proper bundles and instead aggregates in the cytoplasm, a phenotype accompanied by endomembrane distribution defects [141]. Notably, treatment with phalloidin, which stabilizes actin filaments, can partially rescue the salt hypersensitivity of *rsa3-1* mutants [141]. This finding underscores the importance of maintaining proper actin organization to minimize ROS-induced damage and suggests that cell wall integrity, actin dynamics, and ROS homeostasis are mechanistically linked in salt adaptation.

Despite the established reciprocal relationship between ROS and plant actin dynamics, critical knowledge gaps remain. The specific redox-sensing cysteine residues of most plant ABPs, as well as tissue- and stress-specific downstream signaling cascades linking redox modification to actin remodeling, have not been fully elucidated and require further experimental validation. The most tractable starting point is to focus on ABPs with conserved, surface-exposed cysteine residues that are known to be redox-modified in other systems, such as ADF/cofilin and capping protein, and to test whether their oxidation status changes specifically under salt stress using redox proteomics combined with site-directed mutagenesis.

### 5.4. ABA Signaling Cross-Talk with Actin Remodeling

ABA, the quintessential stress hormone, accumulates rapidly under salt stress and mediates numerous adaptive responses, including stomatal closure, osmotic adjustment, and growth inhibition. Emerging evidence indicates that ABA signaling intersects with actin cytoskeletal dynamics through multiple mechanisms, adding another layer of regulatory complexity to salt-induced actin remodeling.

The core ABA signaling cascade includes PYR/PYL/RCAR receptors, PP2C phosphatases, and SnRK2 kinases, with downstream signaling converging on ABF/AREB bZIP transcription factors that govern the transcription of ABA-inducible genes. One prior study identified a functional link between ADF5 and ABA-dependent stomatal movement. Loss-of-function *adf5* mutants exhibited accelerated water transpiration in detached leaves, reduced survival under drought treatment, and impaired ABA-triggered stomatal closure, which arises from the compromised F-actin bundling capacity of ADF5 and consequent disordered actin remodeling [86]. Subsequent molecular assays confirmed that this regulatory axis is strictly ABA-dependent. Mechanistically, the ABF/AREB transcription factor DPBF3 directly associates with the ABA-responsive element (ACGT/CABF) motif within the *ADF5* promoter to activate its transcriptional expression. Similar to *ADF5*, the actin-bundling protein *FIMBRIN2* (*FIM2*) gene is transcriptionally upregulated by ABA and functions to promote stomatal closure via the regulation of actin cytoskeletal remodeling [142]. SCAB1 is another plant-specific actin-binding and bundling protein that plays a central role in stomatal movement. A recent study revealed that SCAB1 functions as a molecular switch that integrates temporally sequential Ca^2+^ and ABA signals during osmotic stress-induced stomatal closure [131]. In the early phase, CPK3/CPK6 phosphorylate SCAB1, suppressing its F-actin binding affinity and promoting F-actin depolymerization to initiate stomatal closure. As the stress persists, ABA signaling attenuates CPK3 kinase activity and SCAB1 phosphorylation levels, thereby facilitating actin filament reassembly and the completion of stomatal closure [131]. However, whether this CPK3/CPK6-SCAB1 phosphorylation module operates under NaCl stress, rather than osmotic stress alone, remains to be directly tested. Although the molecular functions of most ABPs remain incompletely characterized, accumulating observations across multiple species that ABA regulates the transcription of ABP genes, such as *OsADF3* [87] and *GhVLN4* [95], further support the model that ABA signaling transcriptionally modulates actin dynamics via ABF-mediated induction of ABP-encoding genes, thereby coordinating cytoskeletal rearrangement and stomatal closure under osmotic and saline stress.

ABA influences actin dynamics not only at the transcriptional level but also through post-translational mechanisms involving signaling lipids and calcium. For instance, in guard cells, ABA increases phosphatidylinositol 3-phosphate (PI3P) and phosphatidylinositol 4-phosphate (PI4P) levels, and these phosphoinositides directly activate actin-binding proteins, such as SCAB1, to drive actin remodeling during stomatal closure [143]. Specifically, PI3P binding inhibits SCAB1 oligomerization and modulates its F-actin bundling activity, thereby enabling the dynamic reorganization of actin filaments [104]. In parallel, the ABA-triggered phosphoinositide signaling pathway generates inositol trisphosphate (IP_3_), which releases Ca^2+^ from intracellular stores. The resulting calcium signature acts post-translationally on actin-binding proteins, for example, through CPK-mediated phosphorylation of SCAB1, to coordinate the sequential depolymerization and reassembly of actin filaments required for stomatal closure [104].

The complexity of ABA-actin cross-talk is further underscored by antagonistic interactions with other regulators. CONSTANS (CO), a photoperiodic flowering regulator, physically interacts with ABF1, ABF2, ABF3, and ABF4 and antagonizes their transcriptional regulation functions during salt stress under long-day conditions [144]. CO expression decreases in response to salinity treatment, and CO negatively mediates salinity tolerance by suppressing salinity-responsive gene expression and interfering with ABF3-mediated transcriptional activation. While direct evidence linking CO to actin remodeling is lacking, the antagonistic CO-ABF interaction provides a regulatory node that could modulate ABF-dependent transcriptional control of actin-related genes.

The interplay between ABA signaling and actin dynamics also intersects with cell wall integrity. Salt-adapted cells exhibit morphological changes accompanied by coordinated alterations in cytoskeleton and cell wall biogenesis proteins, many of which are responsive to ABA [12,145]. Actin-related proteins such as AtACT7 and ADF3 are upregulated in salt-adapted cells, and these changes are associated with ABA-responsive gene expression [12]. The physical interaction network analysis indicates that cytoskeleton-associated proteins, including actin filaments and microtubules, represent the largest functional cluster among salt-responsive proteins, highlighting the central role of cytoskeletal reorganization in plant adaptation to salinity, a process intimately connected with ABA signaling.

In summary, salt-triggered actin remodeling is governed by interconnected upstream signalling cascades, encompassing the Ca^2+^-SOS module, CPK-mediated protein phosphorylation, bidirectional ROS-actin feedback, and ABA-dependent regulatory networks. Genetic studies suggest that functional actin reorganization functions in association with the SOS pathway to support salt tolerance, although the precise relationship between the two and the direct ABP targets of SOS kinases remain to be established. CPKs are poised to transduce calcium signals via post-translational modification of actin-regulatory proteins, although their salt-specific substrates are largely uncharacterized. ROS exerts concentration-dependent regulation on actin, while perturbed actin homeostasis in turn modulates ROS production, creating a reciprocal feedback loop. ABA shapes actin dynamics both transcriptionally through ABF-controlled ABP expression and post-translationally via phosphoinositide-calcium signalling. Collectively, these pathways operate in concert rather than in isolation to achieve spatiotemporally controlled actin rearrangement under salt stress, although many direct molecular connections and signal-specifying mechanisms await experimental validation. With these upstream signalling events described, we next turn to downstream physiological consequences of actin remodeling and the functional cross-talk between actin filaments and microtubules under salinity.

## 6. Downstream Physiological Functions of Actin Remodeling in Salt Tolerance

The upstream signaling cascades elaborated above collectively transduce salt stress cues into dynamic actin cytoskeleton rearrangements. Correspondingly, these actin remodeling events mediate multiple salt-adaptive physiological outcomes that have been partially covered in the preceding signaling section. The Ca^2+^-SOS-actin axis serves as a core regulatory module specifically activated under saline conditions, whereby actin cytoskeleton dynamics stabilize cellular ion homeostasis by facilitating the plasma membrane localization and activity of SOS transporters, thereby preventing toxic Na^+^ overaccumulation and maintaining a favorable K^+^/Na^+^ ratio for salt tolerance. Bidirectional crosstalk between actin filaments and Ca^2+^ signals precisely shapes the spatiotemporal patterns of calcium oscillations under salt stress, which enables plant cells to finely calibrate the intensity and duration of downstream salt stress signal outputs and avoids aberrant or exaggerated stress signaling activation. The salt-triggered ROS-actin feedback loop tightly restricts excessive ROS bursts and accumulation in stressed cells, which sustains robust antioxidant enzyme activity and alleviates salt-induced oxidative damage to membrane lipids, proteins, and the photosynthetic apparatus. In addition, ABA–actin signaling interplay is substantially enhanced under salinity, which dynamically modulates stomatal aperture closure to reduce transpiration water loss and maintain leaf water potential during salt-induced osmotic stress. Beyond these signal-coupled physiological outputs, actin reorganization governs several additional core adaptive processes, including plastic root growth and halotropic tropisms, vesicle trafficking for membrane and cell wall preservation, and the repression of salinity-triggered premature programmed cell death, which are elaborated separately below.

### 6.1. Actin Remodeling Drives Plastic Root Growth and Halotropic Responses Under Salinity

Salt stress induces significant and dose-dependent remodeling of root system architecture (RSA), with morphological alterations typically manifesting more severely in roots than in shoots, as roots are the primary organs that perceive soil salinity [146].

A wealth of studies, primarily conducted on staple crops and model plants such as rice, wheat, and *Arabidopsis*, has systematically documented the inhibition of primary root elongation, which is frequently accompanied by a reduction in both the number and length of lateral roots under saline conditions [146,147,148]. In wheat, increasing NaCl concentrations from 50 to 200 mM progressively restrain root elongation [149]. For *Arabidopsis*, moderate to high salinity (75–150 mM NaCl) simultaneously inhibits the elongation of primary roots (PR) and lateral roots (LR), resulting in consistent reductions in PR length, LR length and LR count. Notably, reported LR density responses to severe salt treatments vary considerably among independent studies, a discrepancy generally attributed to divergent nutrient supply regimes adopted in different experimental setups [150,151,152,153]. Under extreme salinity (≥200 mM NaCl), viability assays reveal newly emerged LRs suffer far less injury than elongated LRs and primary roots, yet the physiological relevance of this observation remains unclear given that such high NaCl doses are lethal to seedlings [154]. Conversely, low NaCl levels (<50 mM) stimulate LR outgrowth when 4–5-day-old *Arabidopsis* seedlings are transferred to saline media; this response is salt-specific and absent under equivalent osmotic stress [150,153]. However, no LR developmental alterations are detected if seeds germinate directly on 30 mM NaCl plates, indicating that plant developmental stage dictates distinct root adaptive programs to salinity [152]. While the stage-dependent dynamic regulation of root system architecture (RSA) under salt stress remains largely uncharacterized at the molecular and cellular levels, the developmental stage and salt dosage collectively act as two decisive determinants shaping plant root adaptive morphology in saline environments.

Root systems represent the primary sensory and responsive organs for salt detection, and their adaptive developmental plasticity is largely governed by dynamic actin cytoskeletal rearrangement under saline conditions. Existing evidence confirms a salt dose-dependent pattern of actin rearrangement in plant roots, where lower concentrations (e.g., 50 mM NaCl) induce mild reorganization while higher concentrations (e.g., 140 mM NaCl) trigger drastic microfilament disassembly in the *Arabidopsis* root elongation zone [80,99]. Such salt-induced cytoskeletal shifts arise from altered expression and activity of actin and ABPs, which further interconnect with multiple hormone signaling cascades to shape root system architecture. For instance, in sunflower seedlings, NaCl stress impairs the ordered assembly of ACT8, a vegetative actin isoform expressed in primary root vascular tissues [155]. Such actin disassembly coincides with dissipation of the acropetal PIN1 auxin efflux carrier gradient within the same cell population. Concurrent disruption of ACT8 architecture and PIN1 polar distribution jointly suppresses primary root elongation and diminishes lateral root outgrowth [155]. The study thus provides direct evidence for a causal link between salt stress-induced actin remodeling and root system architecture restructuring, revealing a probable crosstalk among serotonin, auxin, and actin signaling pathways during the salinity response. A recent study on pigeon pea uncovered a melatonin-centered regulatory module balancing actin turnover and salt tolerance. Melatonin upregulates the kinase CcARP1, which physically interacts with and phosphorylates the actin-severing protein CcADF9 to suppress its filament-disassembling activity. This modification stabilizes F-actin, preserves root elongation, and improves plant salt resistance, whereas overexpressing unmodified CcADF9 exacerbates salt hypersensitivity via excessive actin depolymerization [156].

Beyond general root growth regulation, actin dynamics are essential for salt-induced root hair plasticity, a critical adaptive strategy that increases root surface area for water and nutrient acquisition under salt-limited growth conditions. Proper actin turnover sustains continuous root hair initiation, tip growth, and morphological stability, whereas disrupted actin assembly results in stunted, swollen, or collapsed root hairs, further compromising plant salt adaptability. Root hair developmental plasticity in response to salinity stress involves both transcriptional reprogramming and actin cytoskeletal dynamics. Early work established that salt stress inhibits root hair elongation and density in a dose-dependent manner, an effect driven primarily by ionic disequilibrium rather than osmotic stress [157,158]. Notably, salt stress disrupts the normal root epidermal cell pattern by altering the expression of the homeobox transcription factor GLABRA2 (GL2), which determines hair versus non-hair cell fate, leading to cell fate switches and a substantial reduction in root hair number. These plastic responses are mediated at least in part by the SOS pathway and appear to represent an adaptive strategy to limit excessive Na^+^ uptake under saline conditions. Building upon this framework, a recent comprehensive review [159] synthesizes current understanding of the molecular mechanisms underlying salt-induced root hair plasticity, highlighting that salinity interferes with actin dynamics, Ca^2+^ gradients, and the expression of key transcription factors such as RHD6 (Root Hair Defective 6) and RSL4 (Root Hair Defective 6-Like 4) to suppress root hair growth.

Most importantly, actin cytoskeleton remodeling acts as a core driving factor for plant halotropism, the directional root growth response that enables roots to avoid high-salinity microenvironments. Asymmetric actin polymerization and depolymerization establish differential growth patterns across root flanks, which further modulates polar auxin distribution and redirects root growth away from salt-inhibited regions [155,160,161]. It was demonstrated that light exposure alters root sensitivity to salinity and affects halotropic responses [161]. In *Arabidopsis*, illumination of roots promotes root growth through a ROS-mediated and F-actin-dependent process [161]. Conversely, keeping roots in darkness alters F-actin distribution, polar localization of PIN auxin transporters, and polar auxin transport. This interplay between light signaling, actin organization, and auxin transport directly influences the root’s ability to navigate away from saline patches in the soil. Light-exposed roots were found to be less effective in their salt-avoidance behavior, implying that actin dynamics modulated by environmental cues govern the directional growth responses that optimize root exploration in heterogeneous saline soils.

### 6.2. Actin-Dependent Vesicular Trafficking Maintains Membrane and Cell Wall Integrity

Salt stress imposes severe osmotic and mechanical stress on plant cells, triggering plasmolysis, membrane lipid peroxidation and irreversible damage to cell wall architecture [162]. As the primary intracellular track for endomembrane transport, dynamically remodeled actin filaments coordinate exocytosis and endocytosis to safeguard plasma membrane integrity and reinforce cell wall structures under saline environments. While myosin-driven vesicle movement relies on ordered cortical actin arrays, the evidences that chemical depolymerization of actin with LatB severely blocks cargo delivery and exacerbates salt-induced membrane breakdown confirms actin remodeling as an indispensable prerequisite for functional vesicle trafficking under salinity [163,164].

Exocytic transport guided by actin filaments delivers multiple protective cargoes to the cell periphery during salt acclimation. Vesicles loaded with cellulose synthase complexes (CSCs), pectin precursors, and lignin monomers move along actin cables from the trans-Golgi network to the plasma membrane, sustaining continuous cell wall biosynthesis and cross-linking [165]. Pharmacological disruption of the actin cytoskeleton using latB alters Cellulose Synthase A (CESA) density and distribution at the plasma membrane and inhibits Golgi motility, demonstrating that actin tracks are essential for proper cellulose synthase delivery [166,167]. Mechanically, myosin XI and the actin cytoskeleton coordinate the exocytosis of CESA-containing compartments; disruption of myosin activity reduces the delivery rate of CSCs to the plasma membrane and increases failed vesicle secretion events [69].

Recent research has revealed functional diversification among myosin XI members in stress responses. While all three major myosin XI isoforms—XI-K, XI-2, and XI-1—contribute to cytoplasmic streaming under normal conditions and their expression is induced upon salt exposure [21], only the loss-of-function mutant of *AtXI-1* exhibits enhanced salt tolerance. Specifically, *atxi-1* mutants accumulate less Na^+^, maintain higher chlorophyll and proline contents, and show reduced oxidative damage compared to wild-type plants under salt stress, suggesting that myosin XI-1 negatively regulates salt tolerance by modulating intracellular Na^+^ homeostasis. Notably, this salt-tolerant phenotype is also observed in the *XI-K/XI-2/XI-1* triple mutant, whereas mutants of *XI-K* or *XI-2* alone, as well as the *XI-K/XI-2* double mutant, show no significant difference from the wild type, underscoring the specific and non-redundant role of myosin XI-1 in this response [21]. Intriguingly, the triple mutant exhibits reduced seed germination under salt stress, suggesting a stage-specific and potentially complex regulatory mechanism that warrants further investigation.

The actin-dependent secretion reinforces cell wall rigidity, reduces plasmolysis severity, and mitigates mechanical rupture under high osmotic pressure. Actin cytoskeleton organization is clearly affected in *Arabidopsis* root cells exposed to salt stress, and disruption of actin dynamics impairs cell survival under saline conditions. Meanwhile, actin tracks mediate the surface trafficking of ion transporters including SOS1 and aquaporins such as PIP2;7, ensuring their proper plasma membrane localization to maintain cellular ion and water balance. Sorting Nexin 1 (SNX1) co-localizes and interacts with SOS1 in endosomes, promoting its plasma membrane localization and protein stability under saline conditions; salt-induced SOS1 phosphorylation enhances this interaction, which is required for SOS1 PM localization [168]. Furthermore, selective aquaporin isoforms related to the stress response, including PIP2;7, are found in detergent-resistant membrane microdomains, and this protein partitioning may act as a mechanism to regulate aquaporins involved in the response to salt stress [169].

In parallel, actin-supported endocytic pathways clear damaged and oxidized membrane components generated by salt-triggered ROS burst. Endosomes carrying impaired lipid and protein cargo travel along actin filaments for degradation or recycling, a process supported by evidence that actin organization and endocytosis are functionally linked to salt stress responses in yeast [170]. This clearance mechanism prevents toxic debris accumulation that would otherwise disrupt membrane fluidity and permeability homeostasis. Actin also modulates the trafficking of R-SNARE proteins such as VAMP714, which mediate fusion events between endosomes and vacuoles. Loss of VAMP714 function disturbs actin-dependent cargo sorting and elevates cytoplasmic H_2_O_2_ levels, aggravating salt-induced oxidative injury. Recent studies demonstrate that VAMP714 is a stress-inducible trafficking regulator whose deficiency leads to aberrant H_2_O_2_ accumulation, with mutant plants showing enhanced ROS accumulation and compensatory upregulation of detoxification pathways [171,172]. Transcriptome analysis of *vamp714* mutants revealed early changes in redox and vesicle trafficking genes, confirming that VAMP714-mediated vesicle fusion is integral to ROS compartmentalization and redox regulation under salinity [172].

Beyond plasma membrane turnover, actin-linked vacuolar vesicle transport facilitates compartmentalization of excess Na^+^ via NHX antiporters packed in Golgi-derived vesicles. Cryo-NanoSIMS imaging has demonstrated that under moderate to high salt stress, *Arabidopsis* root meristem cells switch from sodium accumulation in cell walls to vacuolar sequestration as the dominant detoxification strategy, with SOS1 (a plasma membrane-type NHX) also accumulating at late endosomes/prevacuoles and vacuoles, supporting its role in vacuolar sodium sequestration [173]. Meanwhile, vacuolar-type NHX proteins (e.g., OsNHX1) function as primary transporters for Na^+^ compartmentalization into vacuoles, maintaining cytosolic Na^+^ at low levels and alleviating ionic toxicity [174]. This vacuolar sequestration mechanism, coordinated with the actin-trafficking machinery, represents a critical adaptive strategy for maintaining ion homeostasis under saline conditions.

Actin-vesicle crosstalk also governs adaptive structural adjustments in specialized salt-tolerant cell types, such as the salt glands of recretohalophytes [175,176]. Fluorescence labeling studies have revealed abundant actin filaments surrounding secretory vesicles in salt gland secretory cells, where actin bundles drive the transport of ion-laden vesicles toward the cuticular chamber for salt extrusion [177]. Pharmacological inhibition of actin polymerization suppresses vesicle fusion with the plasma membrane and drastically reduces salt secretion efficiency, indicating that the actin cytoskeleton plays a conserved and essential role in vesicle-mediated salt excretion in halophytic species [178]. These findings align with broader evidence that actin dynamics regulate vesicle trafficking and secretion across diverse secretory systems [179], underscoring the functional significance of actin-membrane interactions in plant adaptation to salinity.

### 6.3. Actin Dynamics Repress Salt-Induced Premature Programmed Cell Death

Severe or prolonged salt stress triggers irreversible cellular damage and initiates premature PCD, which impairs root tissue viability and restricts plant growth and survival [82]. Emerging evidence has established dynamic actin cytoskeleton remodeling as a crucial protective mechanism that actively represses salt-triggered PCD and sustains cellular viability under saline conditions [13]. Appropriate actin turnover, rather than excessive actin bundling or complete depolymerization, serves as a key cellular checkpoint to avoid stress-induced lethal damage [93]. Under normal salt acclimation, tightly regulated actin rearrangement buffers exaggerated stress signaling, including aberrant calcium spiking and excessive ROS accumulation, two upstream triggers that act synergistically to activate plant PCD pathways [83].

Defective actin remodeling caused by loss-of-function of core actin-binding proteins severely disrupts this protective mechanism. For instance, *prf5* mutants display disordered actin architecture under salt and osmotic stress, accompanied by exaggerated calcium signaling and excessive ROS accumulation, which eventually trigger premature PCD in root elongation zones [99]. Consistently, pharmacological actin depolymerization with LatB reproduces this stress-hypersensitive, cell death-prone phenotype, establishing a causal link between intact actin homeostasis and PCD inhibition. Mounting evidence indicates this regulatory axis relies heavily on mitochondria and the associated calcium signaling. Loss of functional Arp2/3 complex in *arp2* mutants impairs actin nucleation, which in turn amplifies salt-triggered calcium signals, induces mitochondrial clustering, activates the mitochondrial permeability transition pore (mPTP), and dissipates mitochondrial membrane potential, all hallmark events that drive stress-induced PCD [93]. Genetic and pharmacological assays further verify that blocking mPTP opening or attenuating calcium overloading partially rescues the salt-sensitive defects of *arp2* seedlings, highlighting actin-dependent control of mitochondrial calcium flux as a vital brake on premature cell death. Studies on mung bean (*Vigna radiata*) cotyledons have uncovered an intramitochondrial actin pool, whose dynamic turnover governs cytochrome c release—the rate-limiting step of PCD onset [180]. Application of actin-depolymerizing compounds lowers mitochondrial membrane potential and accelerates cytochrome c efflux, directly proving that compromised mitochondrial actin integrity acts as a trigger for cell death progression.

Similar to other actin-mediated physiological regulations, most mechanistic models linking actin dynamics to PCD inhibition are primarily established based on osmotic stress phenotypes. Considering that salt stress shares conserved downstream cellular stress responses and PCD regulatory pathways with osmotic stress, it is reasonable to propose that actin-dependent suppression of premature cell death represents a vital adaptive strategy for plants to cope with high-salinity environments.

In summary, actin cytoskeleton remodeling triggered by salt stress drives a suite of downstream adaptive physiological responses. Fine-tuned actin dynamics assist ion homeostasis by mediating the trafficking and membrane targeting of ion transporters such as SOS1. Actin also mediates root plastic development under salinity, regulating primary-root elongation, lateral-root formation, root-hair development and halotropic avoidance, in coordination with auxin and light signals. Actin-myosin-based vesicle trafficking supports cell-wall biosynthesis, clearance of impaired membrane constituents, vacuolar sodium compartmentalization, and salt excretion in halophytes. Critically, appropriate actin turnover serves as a cellular checkpoint to suppress salt-induced premature programmed cell death by constraining deleterious calcium over-spiking, ROS accumulation and mitochondrial cell-death cascades. Several regulatory models are partially derived from osmotic-stress assays and still require further salt-specific experimental verification. Taken together, these diverse physiological consequences demonstrate how the actin cytoskeleton converts upstream salt-stress signalling into multi-layered cellular adaptation, positioning actin remodeling as a central determinant of salt-tolerance outcomes. With these actin-dependent physiological responses established, we next discuss the reciprocal cross-talk between actin filaments and microtubules under salt-stress conditions.

## 7. Cross-Talk Between Actin Filaments and Microtubules Under Salt Stress

Traditionally investigated as separate cytoskeletal structures, actin filaments and microtubules operate via tightly coordinated dynamics to support plant salt tolerance, with their crosstalk manifested through physical association, shared regulatory modules, and distinct yet complementary physiological functions. Recent work has begun to systematically map these interactions during plant development, revealing that AF and MT arrays are connected through dual-function proteins, shared signaling components, and coordinated remodeling [181].

Live-cell dual-fluorescence imaging using rice cytoskeletal reporter lines offered direct visual evidence of their physical linkage under salinity. Time-lapse tracking of rice root epidermal cells exposed to 250 mM NaCl revealed that microtubules undergo a cycle of depolymerization and reassembly after salt exposure, relying on transient microtubule foci as assembly templates [182]. Notably, these foci require intact actin filaments for proper trafficking and positioning. Pharmacological actin depolymerization with latB drastically delays microtubule repolymerization after salt damage, confirming actin acts as a structural scaffold to restore microtubule arrays. This actin-dependent facilitation of microtubule recovery emphasizes an important function of the actin cytoskeleton during salt stress, potentially by maintaining cell activity such as trafficking during salt exposure. Beyond this scaffold function, direct physical coupling between the two networks is mediated by dual-function proteins that simultaneously bind actin filaments and microtubules, such as kinesin-like calmodulin-binding protein (KCBP) and certain plant formins [181,183,184]. These proteins provide molecular bridges that coordinate the spatial arrangement of the two arrays, although their specific roles under salt stress remain to be directly demonstrated.

The prefoldin (PFD) complex represents a core shared regulator coordinating the biogenesis of both actin and tubulin. Genetic analysis of *Arabidopsis pfd3* and *pfd5* mutants demonstrated compromised tubulin accumulation and disorganized microtubules under salt stress, leading to ion-specific NaCl hypersensitivity (unchanged sensitivity to mannitol or LiCl) [185]. Functional complementation assays in yeast further verified the conserved salt-protective function of plant PFD subunits, highlighting this chaperone complex as an indispensable shared checkpoint maintaining dual cytoskeleton integrity under ionic stress. This finding demonstrates that the coordinated folding and assembly of both actin and tubulin, mediated by the prefoldin complex, are essential for the plant’s capacity to withstand salt stress, highlighting a shared regulatory mechanism that coordinates the integrity of both cytoskeletal systems.

Some other pharmacological assays uncovered divergent, mutually supportive roles for the two cytoskeletons during salt acclimation. Stabilizing actin with phalloidin improves *Arabidopsis* salt survival, while actin depolymerizing compounds (LatA, cytochalasin D) aggravate salt injury [79,186]. Conversely, microtubule stabilization via paclitaxel exacerbates salt-induced cell death, whereas microtubule-disrupting agents (oryzalin, propyzamide) enhance tolerance. This functional divergence arises from distinct upstream triggers: actin polymerization responds to both osmotic and ionic signals, while microtubule remodeling is selectively activated by Na^+^ toxicity. Consistently, loss-of-function *sos1*/*sos2* mutants exhibit constitutive microtubule disorder, linking microtubule homeostasis to SOS-mediated ion homeostasis [187].

Phospholipid signaling provides another layer of coordination between actin and microtubule dynamics under salt stress. Phospholipase D (PLD)-derived phosphatidic acid (PA) plays pivotal roles in modulating both cytoskeletal systems. PA binds to and enhances the activity of MAP65-1, a microtubule-associated protein that promotes microtubule polymerization and bundling, thereby improving plant salt tolerance [188]. Concurrently, PA interacts with the heterodimeric capping protein (AtCP) at actin filament ends, inhibiting its capping activity and allowing rapid actin assembly and elongation [189]. This dual regulation of microtubules and actin by the same phospholipid signal suggests a coordinated mechanism wherein PA orchestrates the simultaneous remodeling of both cytoskeletal networks to achieve adaptive responses to salt stress.

A further layer of coordination is provided by ROP GTPase signaling, which operates as a central switch controlling the antagonistic arrangement of actin filaments and microtubules during polarized cell growth [190,191]. In this module, ROP2 activates RIC4 to promote the organization of cortical actin filaments, while simultaneously suppressing RIC1-mediated microtubule rearrangement, thereby establishing a mutually exclusive spatial pattern of the two arrays [190]. Importantly, the ROP2-RIC1 branch has been directly implicated in salt tolerance: salt stress rapidly activates ROP2, which recruits RIC1 from microtubules to the plasma membrane, thereby promoting microtubule reassembly after salt-induced depolymerization [192]. Although the ROP2-RIC4-actin branch has not yet been directly examined under salt stress, its components overlap substantially with salt-responsive signaling, suggesting that a comparable ROP-dependent mechanism may coordinate actin–microtubule dynamics under salinity.

Furthermore, recent studies in other biological contexts have begun to reveal the mechanistic principles underlying actin-microtubule coordination, offering conceptual and technical frameworks that may guide future work on salt-stressed plants. In the male germ unit of pollen, microtubules and kinesins mediate fine positioning and directional control, while the actomyosin system drives oscillatory movement, providing a clear demonstration that the two cytoskeletal systems can adopt distinct yet complementary roles within the same cell [193]. This division of labor, in which each network executes a specialized function rather than acting redundantly, suggests a useful framework for dissecting whether actin and microtubules likewise partition their roles during salt acclimation. In a different context, the bacterial effector RipU physically associates with and disrupts both actin and microtubule organization in tomato, and pharmacological disruption of the cytoskeleton promotes *Ralstonia solanacearum* colonization [194]. Although this study concerns biotic rather than abiotic stress, it demonstrates that a single pathogen-derived factor can coordinately target both cytoskeletal systems and that such coordination is functionally significant for the host response. This raises the possibility that, under salt stress, shared signaling components may similarly act on both actin and microtubules to coordinate their remodeling. Together, these cross-context studies illustrate the value of examining actin-microtubule crosstalk from a comparative perspective, and suggest that mechanistic and technical approaches developed in developmental and biotic-stress systems may be adapted to dissect salt-specific coordination between the two cytoskeletal networks.

In summary, multiple lines of evidence demonstrate that actin and microtubule networks are tightly coupled during salt acclimation through shared signaling hubs and pharmacological coordination, while physical bridges mediated by dual-function proteins such as KCBP and formins have been characterized in developmental contexts but remain to be directly demonstrated under salt stress. Major outstanding questions requiring further experimental investigation include the salt-specific function of the ROP-RIC module and the identification of direct protein linkers that physically bridge actin and microtubules under saline conditions.

## 8. Future Perspectives

Although considerable progress has been made in unraveling actin cytoskeleton-mediated salt tolerance mechanisms in plants, multiple critical gaps remain to be addressed in future studies. Given the evidence reviewed above, we propose that three questions should be prioritized, as they are both experimentally tractable and directly linked to unresolved contradictions in the current literature.

First, do specific actin isoforms exert non-redundant functions under salt stress? Transcriptomic evidence indicates that individual *ACTIN* paralogs display distinct salt-responsive expression patterns, yet their functional non-equivalence remains untested under salinity. A priority experiment is to generate tissue-specific loss-of-function lines for the most salt-responsive isoforms (e.g., ACT7 in *Arabidopsis*) and assess salt tolerance at cell-type resolution. The measurable outcomes include salt-induced changes in actin organization, root growth, and survival rate. If distinct isoforms are functionally non-equivalent, this would justify targeting specific *ACTIN* genes rather than the entire actin family in crop improvement.

Second, does the CPK-ABP phosphorylation module operate under NaCl rather than osmotic stress alone? The CPK3/CPK6-SCAB1 phosphorylation module has been demonstrated under osmotic stress-induced stomatal closure, but its function under NaCl stress remains untested. A priority experiment is to test whether salt-induced stomatal closure requires CPK3/CPK6-mediated SCAB1 phosphorylation, using *cpk3 cpk6* double mutants and phospho-dead or phospho-mimetic SCAB1 variants. The measurable outcomes encompass stomatal aperture dynamics, SCAB1 phosphorylation levels, and F-actin organization in guard cells under NaCl versus mannitol treatments. If the module operates specifically under salt stress, this would establish a direct molecular link from salt perception to actin remodeling. Moreover, whether CPK-mediated phosphorylation extends to other ABPs under NaCl stress remains to be tested.

Third, can manipulating specific actin-regulatory modules improve crop salt tolerance without incurring growth penalties? A priority experiment is to test stress-inducible, tissue-specific manipulation of specific actin-regulatory modules in a target crop. For example, salt-inducible modulation (overexpression or knockdown) of salt-responsive ADF isoforms (e.g., SaADF2 or OsADF3) in rice, or root-specific manipulation of PRF5 homologs, could be tested in field trials. The measurable outcomes should be clearly distinguished: (i) gene-expression changes (transcript abundance of the manipulated gene and downstream stress markers); (ii) tolerance in a heterologous model (e.g., *Arabidopsis* or tobacco); and (iii) performance in the target crop (grain yield under saline field conditions). This three-tier evaluation would determine whether cytoskeleton-focused strategies translate from model systems to crops.

Finally, emerging technologies, including super-resolution live-cell imaging, single-cell omics, and specific cytoskeletal biosensors, will continue to advance our systematic understanding of cell-type-specific and spatiotemporally dynamic actin regulatory networks underlying plant salt tolerance. However, the most urgent need is not additional descriptive characterization but hypothesis-driven experiments that connect specific actin-regulatory modules to measurable salt-tolerance outcomes in crops. While the mechanistic insights summarized here remain far from direct field application, they provide a conceptual foundation that may guide future efforts toward cytoskeleton-informed crop improvement, once the key unresolved questions are addressed.

## 9. Conclusions

This review synthesizes current evidence positioning the actin cytoskeleton as a central integration node, rather than a passive downstream effector, during plant salt acclimation. Three core conclusions emerge. First, the genetic diversity and stress-responsive transcription of actin genes establish a functionally non-equivalent repertoire of actin isoforms; this also cautions against using actin genes routinely as RT-qPCR reference genes under saline conditions. Second, salt-triggered actin remodeling is spatiotemporally heterogeneous and bidirectionally coupled to Ca^2+^-SOS, CPK, ROS, and ABA signaling. Distinct ABP isoforms exert antagonistic or counterbalancing activities, governing the balance between adaptive actin assembly and deleterious depolymerization. Third, actin dynamics are causally linked to salt-tolerance-associated phenotypes including ion homeostasis, plastic root growth, vesicle trafficking, and the inhibition of premature programmed cell death. Furthermore, actin remodeling coordinates with microtubules via shared regulators to fine-tune salt acclimation. Collectively, these findings reframe actin dynamics as a tunable regulatory layer that bridges salt perception and physiological adaptation.

## Figures and Tables

**Table 1:** Summary of ABPs implicated in actin remodeling under salt stress and related stress conditions.

ABPs	Species	Stress or Treatment	Key Findings	Evidence Type *	Ref.
ADF1	*Arabidopsis*	Salt	*adf1* mutants show reduced plant salt tolerance, with increased percentage of actin cables, and reduced actin filament density, whereas *ADF1* overexpression lines display the opposite phenotypes. ADF1 severs/depolymerizes actin filaments to maintain actin organization under salt stress; MYB73 negatively regulates its expression.	D	[85]
ADF5	*Arabidopsis*	Drought; ABA	*adf5* mutants exhibit reduced drought tolerance, and impaired ABA-triggered stomatal closure. DPBF3 (ABF/AREB transcription factor) directly binds the *ADF5* promoter to activate its transcription; ADF5 promotes stomatal closure via F-actin bundling and actin cytoskeleton remodeling in guard cells under ABA and drought stress.	I	[86]
ADF7–VLN1	*Arabidopsis*	Osmotic	Analyses on *adf7* and *VLN1* mutants demonstrate that ADF7 positively and VLN1 negatively regulate root hair formation. ADF7 represses VLN1 expression/function to reduce F-actin bundling, enhance F-actin depolymerization and turnover.	I	[78]
OsADF3	Rice, *Arabidopsis*	Drought; Salt;	OsADF3 is a nuclear actin depolymerizing factor preferentially expressed in vascular tissues; its expression is induced by ABA, salt and drought. Heterologous expression of *OsADF3* in *Arabidopsis* improves drought tolerance, with longer primary roots and upregulated drought-responsive genes.	D (E)	[87]
TaADF16	Wheat, *Arabidopsis*	Cold	Among 25 *TaADF* members, *TaADF13/16/17/18/20/21/22* are markedly responsive to cold acclimation/freezing. Heterologous expression of *TaADF16* in *Arabidopsis* enhances freezing tolerance.	I	[88]
TaADF16	Wheat	Cold; ABA; Salt	*TaADF16* is strongly induced by cold and weakly induced by ABA and NaCl. TaADF16 is phosphorylated during cold acclimation. TaADF-dependent cytoskeleton rearrangement is required for freezing tolerance.	D (E)	[89]
GhADF6	Cotton (*Gossypium hirsutum*)	Alkali	*GhADF6* expression is downregulated under alkaline stress, correlating with increased actin filament stability. Polyacrylic acid (PAA)-coated Mn_3_O_4_ nanozymes (PMO) treatment further suppressed *GhADF6* expression, elevates AF stability and reduces Na^+^ content.	I	[90]
MsADF1/2/3/6/9	Alfalfa (*Medicago sativa*)	Salt; drought	5 out of 9 *MsADF* genes (*MsADF1*, *MsADF2*, *MsADF3*, *MsADF6*, *MsADF9*) are strongly induced by salt and drought, implying their potential roles in abiotic stress resistance.	D (E)	[91]
SaADF2	*Spartina alterniflora*, rice	Salt	Heterologous expression of *SaADF2* (*SaADF2*-OE) in rice improves salt and drought tolerance compared with WT and OsADF2-OE lines; SaADF2-OE maintains intact grana structure and actin organization under dehydration. A serine-6 substitution alters its interaction with OsCDPK6, enhances actin dynamics and F-actin depolymerization capacity to preserve actin organization under stress.	D	[92]
ARP2/3 (ARP2, ARPC5)	*Arabidopsis*	Salt	*arp2-1*, *arp2-2*, and *arpc5* T-DNA insertion mutants exhibit disrupted actin architecture, accelerated F-actin breakdown, mitochondrial dysfunction, elevated Ca^2+^ signaling and salt-triggered PCD with reduced survival; complementation rescues salt tolerance. ARP2/3 complex maintains actin organization under salt stress; actin perturbation induces mitochondrial Ca^2+^ dysfunction and salt-dependent cell death	D	[93]
SPI-BRK1-SCAR/WAVE	*Arabidopsis*	Salt	*spi* mutants and BRK1-overexpressing lines both exhibit salt hypersensitivity in root hairs, and salt-induced actin defects mimic *spi* mutant phenotypes. SPI stabilizes BRK1 to sustain SCAR/WAVE-ARP2/3-dependent actin nucleation under salt stress.	D	[20]
VLN1	*Arabidopsis*	Osmotic; salt	*vln1* mutants have longer root hairs with defective thick actin bundles; *VLN1* overexpression shows opposite phenotypes. VLN1 bundles F-actin in a Ca^2+^/calmodulin-independent manner and protects actin filaments from ADF-mediated depolymerization.	I	[50]
VLN1	*Arabidopsis*	Osmotic; salt	*gl2* mutants share root hair and actin defects with *vln1*, and *VLN1* overexpression rescues *gl2* phenotypes. GL2 directly binds the *VLN1* promoter to activate its expression; VLN1 promotes thick actin bundle formation to terminate rapid root hair elongation under osmotic stress.	I	[78,94]
GhVLN4	Cotton (*Gossypium hirsutum*), *Arabidopsis*	Salt	GhVLN4 is an actin-bundling protein that attenuates stress-induced actin depolymerization to maintain actin bundles under stress. *GhVLN4*-silenced cotton is more susceptible to *Verticillium dahliae*, while heterologous *GhVLN4* expression in *Arabidopsis* improves resistance to *V. dahliae* and tolerance to salt and drought.	D	[95]
GhVLNs	Cotton (*Gossypium* spp.)	Salt; drought; cold; heat	*GhVLNs* promoters contain abundant abiotic-stress and hormone-related cis-elements. Most *GhVLN* genes are expressed in multiple tissues, and several members are highly induced by salt, drought, cold and heat.	D (E)	[96]
OsPRF1, OsPRF2, OsPRF3	Rice	salt, drought, cold, heat, UV	Three rice *OsPRF* genes (*OsPRF1*, *OsPRF2*, *OsPRF3*) localize to cytoplasm and nucleus and bind G-actin. They are differentially regulated by abiotic stresses and hormone treatments; Notably, *OsPRF2* and *OsPRF3* are strongly responsive to NaCl treatment.	D (E)	[97]
BjPRFs	*Brassica juncea*	Heat; drought	23 *BjPRF* genes in *B. juncea* showed differential spatiotemporal expression during plant development and stress conditions.	I	[98]
PRF5	*Arabidopsis*	Salt; osmotic	*prf5* mutants are hypersensitive to NaCl and mannitol treatments, with disordered actin architecture and reduced cell viability. PRF5 restrains salt-induced actin bundling to facilitate orderly actin remodeling; loss of *PRF5* causes abnormal Ca^2+^ oscillations and premature cell death in the root elongation zone.	D	[99]
AtPFN2	*Arabidopsis*	Heat	AtPFN2 acts as a holdase molecular chaperone, preventing heat-induced aggregation of malate dehydrogenase (MDH) in a dose-dependent manner at 43 °C.	I	[100]
AtFH12	*Arabidopsis*	Salt	AtFH12 is a salt-inducible formin, but its mutants show no obvious salt phenotypes, indicating functional redundancy of class II formins. *AtFH12* mutations strongly aggravate the toxic effects of GFP-mTalin and cause abnormal actin structures in *atfh12* × GFP-mTalin lines.	D	[101]
AtFH5	*Arabidopsis*	Salt	*atfh5* mutants are salt hypersensitive. AtFH5-GFP expressed under its native promoter rescues the mutant phenotype. *AtFH5* expression is salt-induced; its actin function is inferred from its identity as an actin nucleator, with no direct measurement of actin dynamics under salt stress	D	[22]
GrFH19, GrFH26	Cotton (*Gossypium raimondii*)	Salt	*GrFHs* promoters contain multiple cis-acting elements associated with development and abiotic stress. *GrFH19* and *GrFH26* are upregulated under salt stress and may modulate cytoskeletal dynamics for salt adaptation.	D (E)	[102]
GhFH20, GhFH30	Cotton (*Gossypium hirsutum*)	Salt; heat	*GhFH20* and *GhFH30* are salt-responsive. Their promoters carry stress-responsive cis-elements, suggesting roles in cytoskeleton-mediated stress adaptation	D (E)	[103]
SCAB1	*Arabidopsis*	Osmotic Stress; ABA	PI3P negatively regulates SCAB1-mediated F-actin bundling by disrupting SCAB1 oligomerization, to facilitate F-actin reorganization during ABA-driven stomatal closure. PI3P-binding-deficient SCAB1 fails to rescue *scab1* phenotypes.	I	[104]

* D, direct salt evidence; D (E), direct salt evidence with salt-responsive expression only; I, inferred evidence from other stresses.

## Data Availability

No new data were created or analyzed in this study. Data sharing is not applicable to this article.

## Abbreviations

The following abbreviations are used in this manuscript:

ABA	abscisic acid
ROS	reactive oxygen species
MT	microtubule
ABPs	actin-binding proteins
ADFs	actin-depolymerizing factors
SOS	Salt Overly Sensitive
ARP2/3	actin-related protein 2/3
RSA3	Short Root in Salt Medium 3
RhoGDI1	Rho GDP dissociation inhibitor 1
ROP2	Rho of plants 2
SPI	SPIRRIG
BRK1	BRICK1
G-actin	globular actin
F-actin	filamentous actin
AFs	actin filaments
NPF	nucleation-promoting factor
Pi	inorganic phosphate
SCAR/WAVE	Suppressor of cAMP Receptor/WASP-family Verprolin-homologous
SRA1/PIR121	SCAR-associated protein 1
Nap1	NCK-associated protein 1
Abi-1/Abi-2	Abl-interactor 1/2
ER	endoplasmic reticulum
PM	plasma membrane
ABD2	actin-binding domain 2
LatA	latrunculin A
LatB	latrunculin B
PCD	programmed cell death
PAA	Polyacrylic acid
VLN	villin
CAP1	cyclase-associated protein 1
PRF	profilin
HMW	high molecular weight
LMW	low molecular weight
FH1	Formin Homology 1
FH2	Formin Homology 2
PTEN	Phosphatase and Tensin Homolog
CPKs	calcium-dependent protein kinases
SCAB1	STOMATAL CLOSURE-RELATED ACTIN BINDING PROTEIN1
OABs	oxidation-induced actin bodies
CP	capping protein
DPI	diphenyleneiodonium
*FIM2*	*FIMBRIN2*
PI3P	phosphatidylinositol 3-phosphate
PI4P	phosphatidylinositol 4-phosphate
IP_3_	inositol trisphosphate
CO	CONSTANS
PR	primary roots
LR	lateral roots
GL2	GLABRA2
RSA	root system architecture
RHD6	Root Hair Defective 6
RSL4	Root Hair Defective 6-Like 4
CSCs	Cellulose synthase complexes
CESA	Cellulose Synthase A
SNX1	Sorting Nexin 1
mPTP	mitochondrial permeability transition pore
PFD	prefoldin
KCBP	kinesin-like calmodulin-binding protein
PLD	Phospholipase D
PA	phosphatidic acid
